# The single-cell transcriptomic atlas iPain identifies senescence of nociceptors as a therapeutical target for chronic pain treatment

**DOI:** 10.1038/s41467-024-52052-8

**Published:** 2024-10-04

**Authors:** Prach Techameena, Xiaona Feng, Kaiwen Zhang, Saida Hadjab

**Affiliations:** Laboratory of Neurobiology of Pain & Therapeutics, Department of Neuroscience, Karolinska Institutet, Stockholm, Sweden

**Keywords:** Pain, Mechanisms of disease

## Abstract

Chronic pain remains a significant medical challenge with complex underlying mechanisms, and an urgent need for new treatments. Our research built and utilized the iPain single-cell atlas to study chronic pain progression in dorsal root and trigeminal ganglia. We discovered that senescence of a small subset of pain-sensing neurons may be a driver of chronic pain. This mechanism was observed in animal models after nerve injury and in human patients diagnosed with chronic pain or diabetic painful neuropathy. Notably, treatment with senolytics, drugs that remove senescent cells, reversed pain symptoms in mice post-injury. These findings highlight the role of cellular senescence in chronic pain development, demonstrate the therapeutic potential of senolytic treatments, and underscore the value of the iPain atlas for future pain research.

## Abstract (web summary)

Chronic pain remains a major clinical challenge with unclear causes and a need for new treatment. Using the single-cell transcriptomics atlas iPain, here the authors show that nociceptor senescence may drive chronic pain and present senolytics as a potential therapeutic approach.

## Introduction

Currently, around 20% of adults suffer from chronic pain, which would correspond to over 1.2 billion people worldwide, making it a global healthcare crisis^1^. While there are treatments available, these often provide only partial relief if at all, and come with adverse side effects and a risk of drug abuse^2,3^. These treatments also center on pain signaling modulation and do not address the underlying molecular and cellular causes of chronic pain. A better understanding of the molecular changes underlying chronic pain progression could enable the development of innovative, non-addictive therapeutic strategies to effectively alleviate chronic pain. Thus, understanding the mechanisms involved in pain chronification have remained crucial for uncovering new therapeutic avenues.

Pain sensation is conveyed peripherally by the activation of pain-sensing neurons, or nociceptors, whose cell bodies reside in the peripheral sensory ganglia (mostly in the dorsal root ganglia, DRG, alongside the spinal cord and in the trigeminal ganglia, TG). Those neurons are of different subtypes that are classified as peptidergic (PEPs), non-peptidergic (NPs), C-Low Threshold mechanoreceptor (C-LTMRs), and a subtype of somatostatin positive neurons (SST)^4–6^. Nociceptors send peripheral nerve endings throughout the whole body, innervating the skin, dura mater, and deep tissue. Centrally, they project within the spinal cord or the pons where the pain signal is transmitted and integrated locally and sent to upper brain centers where it is perceived as a pain sensation.

The introduction of single-cell RNA sequencing (scRNA-seq) has enabled the transformative advancement in our understanding of the intricate functional diversity of disorder development much like the comprehensive atlases created in other fields such as cancer^7^ and neurological disorders^8^. In a substantial effort to create atlases with further gene coverage than already available and with chromatin dynamic information, we integrated diverse single cell (sc) or single-nuclei (sn) RNA-seq from peer-reviewed datasets with our in-house datasets^5,9–17^ (Table 1), with the aim to further unveil the molecular signatures governing the development and persistence of the different types of chronic pain while also focusing on uncovering a shared mechanism among diverse pain models. With our somatosensory atlases called iPain available on CELLxGENE browser, we have established a foundational framework that builds upon and extends previous research efforts^5,11^ and is crucial for investigating the development of chronic pain, when originating in the first anatomical relay of pain pathway, either in the DRG (iPainDRG) or in the TG (iPainTG). Using iPainDRG, our results identify the senescence of a subset of nociceptor cells as a common mechanism across the various rodent pain models, as well as in humans with chronic pain. Our data further reveal that treatment with senolytic compounds rescues hyperalgesia and allodynia associated with chronic pain in mice, demonstrating significant therapeutic potential and a role for the senescence process in chronic pain disorders independent of aging.

**Table 1:** The table indicating the data sources, single-cell/nuclei technologies, Gene Expression Omnibus accession numbers, and tissues included in iPain

Source	Method	GEO	Tissue
Renthal et al.^5^	snRNA-seq (inDrops)	GSE154659	DRG
Wang et al.^9^	scRNA-seq (10×)	GSE155622	DRG
Avraham et al.^10^	scRNA-seq (10×)	GSE158892	DRG
Zhang et al.^11^	scRNA-seq (10×)	GSE216039	DRG
Sharma et al.^12^	scRNA-seq (10×)	GSE139088	DRG
Parpaite et al.^13^	scRNA-seq (SS2/Patch)	GSE168032	DRG
Techameena_Multi	snMulti-omics (10×)	GSE253345	DRG
Techameena_SS3_DRG	snRNA-seq (SS3)	GSE253345	DRG
Yang et al.^14^	snATAC-seq (10×)	GSE197289	TG
Yang et al.^14^	snRNA-seq (10×/inDrops)	GSE197289	TG
Jia et al.^15^	scRNA-seq (BD)	GSE213105	TG
Liu et al.^17^	scRNA-seq (BD)	GSE186421	TG
Nguyen et al.^16^	scRNA-seq (DropSeq)	GSE101984	TG
Techameena_SS3_TG	snRNA-seq (SS3)	GSE253345	TG

## Results

### Creation of iPain, a somatosensory atlas that captures chronic pain development

**Fig. 1:**
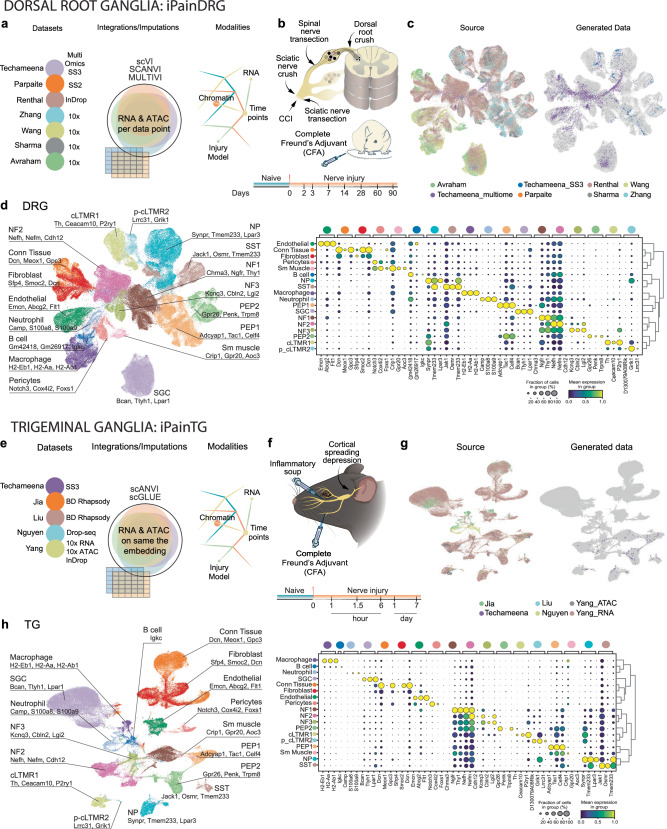
iPain: an integrated atlas of somatosensory changes across diverse neuropathic pain models. **a** A schematic representing the data sources, data modalities, and integration models applied to construct iPainDRG. **b** A schematic to visualize various types of injury models and timepoints included in iPainDRG. **c** UMAP plots of iPainDRG indicating various datasets from different sources (left), and with data from our lab highlighted (right). **d** Left, a UMAP plot of of iPainDRG atlas colored by 18 different cell types predicted from the scANVI model, and cell types were color-coded as seen in a dot plot on the right. Right, a dot plot representing the top 3 gene markers for each cell type. **e** A schematic representing the data sources, data modalities, and integration models applied to construct iPainTG. **f** A schematic to visualize various types of injury models and timepoints included in iPainTG. **g** UMAP plots of iPainTG indicating various datasets from different sources (left), and with data from our lab highlighted (right). **h** Left, a UMAP plot of iPainTG atlas colored by 18 different cell types predicted from scANVI model and cell types were color-coded as seen in a dot plot on the right. Right, a dot plot representing the top gene markers for each cell type. **f** Mouse head and TG icons were created with BioRender.com released under a Creative Commons Attribution-NonCommercial-NoDerivs 4.0 International license.

In recent years, numerous efforts have been made to generate sc/snRNA-seq datasets of the mouse DRG and TG in diverse models of chronic pain (Fig. 1; Supplementary Data 1). These datasets offer different sampling timepoints and pain models at various stages of progression, providing the opportunity to create comprehensive atlases. However, the lack of alignment among the unique genes and molecules identified in each dataset due to the use of different isolation methods and sequencing technologies can be a challenge for integration of different datasets. To overcome this, we have used variational autoencoder (VAE)-based integration models like scANVI^18^, MULTIVI^19^, and scGLUE^20^ (Fig. 1; Supplementary Fig. 1; Supplementary Fig. 2; Supplementary Fig. 3), which can effectively harmonize the datasets, control for batch correction while conserving the biological information to capture the entire spectrum of molecular dynamics and diversity associated with pain chronicity in the DRG and TG in different chronic pain models and at different timepoints (Fig. 1b, f; and Supplementary Fig. 3). The iPainDRG atlas, which integrates 191,798 cells from 7 sources (in-house generate: 4468 cells from multiome and 625 cells from Smart-seq3), represents a comprehensive resource for understanding the molecular dynamics in somatosensory neurons in response to injury and the progression of pain chronification, with RNA and ATAC information provided for each single cell (multiomic dataset allow for the use of MultiVI with ATAC imputation to all cells even to cells that did not originally provide ATAC modality, Supplementary Fig. 2a for workflow, Supplementary Fig. 3a for atlas content). Of note, the combination of all these modalities allows the use of integration methods compatible with the imputation of missing genes, therefore increasing the gene coverage. Similarly, the iPainTG atlas, which integrates 87,838 cells from five diverse sources (in-house generated: 124 cells from Smart-seq3), provides insights into the TG’s response to various pain conditions (unlike in iPainDRG, not every cell in iPainTG has ATAC modality, Supplementary Fig. 2b and Supplementary Fig. 3b). These atlases, with their detailed cell-type-specific markers and chromatin accessibility profiles, can serve as valuable references for the research community studying the underlying mechanisms of chronic pain (Fig. 1d, h; and Supplementary Data 2–3). Finally, the atlases allow for the integration of datasets sequenced by any sequencing technologies.

### Cell state progression towards chronic pain and driving mechanism

**Fig. 2:**
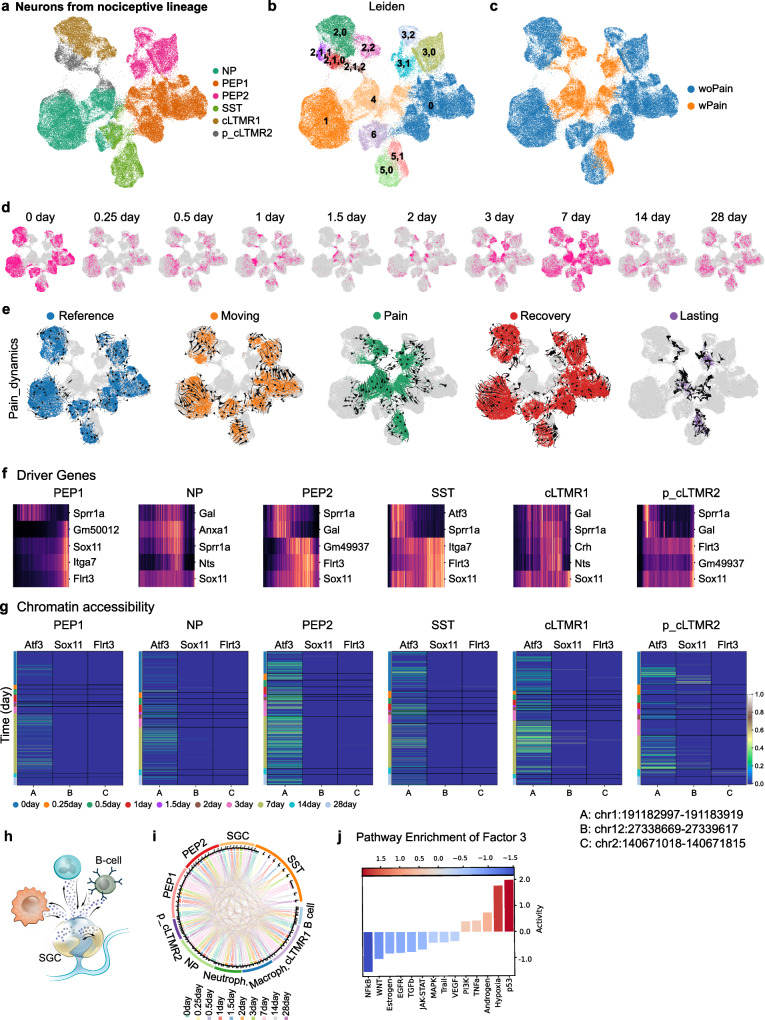
Temporal molecular dynamics of nociceptors and pain states during neuropathic pain. **a** A UMAP plot of the nociceptive lineage cells from iPainDRG colored by their respective subtypes. **b** A UMAP plot of the nociceptive lineage cells highlighted by Leiden clusters. **c** A UMAP plot of the nociceptive lineage cells highlighted by the pain state identified by aggregation of Leiden clusters. **d** UMAP plots of the nociceptive lineage cells highlighted in pink by actual injury timepoints. **e** UMAP plots of the nociceptive lineage cells colored by different states in the pain dynamics with embedded velocity arrows from CytoTrace. **f** Heatmaps representing the expression of top 5 driver genes per nociceptive subtypes as a function of pseudotime. **g** Heatmaps showing the chromatin accessibility of peaks corresponding to the promoter region of *Atf3*, *Sox11*, *Flrt3* genes as actual injury timepoints progressed. The ATAC peak regions are shown on the lower right. **h** A schematic of cell-to-cell communication between nociceptors and immune cells. **i** Chord diagram indicating the communication intensity between subtypes of nociceptors, immune cells, and satellite glia at different injury timepoints. **j** A bar plot of pathways being enriched in Factor 3, identified to be the factor with the most differences in terms of context loading between *Reference* and *non-Reference* (*Moving*, *Pain*, *Recovery*, and* Lasting* combined) cells.

To better understand the progression of cell state changes in sensory neurons during chronic pain, we used the iPainDRG dataset, as it includes more pain models than iPainTG and covers the whole-time course of pain chronification. Indeed, all pain models used in the dataset are known to develop within 1–3 days, peak at 2–3 weeks, and persist for weeks thereafter^21^. Therefore, we chose to cover the analysis of the study up to 28 days post-injury, where the pain is chronic and well established, and in the atlas, several models share 28-day timepoints presumably reinforcing the analysis. Using iPainDRG, we further isolated and re-clustered neurons from the nociceptive lineage in DRG, then visualized with Uniform Manifold Approximation and Projection (UMAP) to examine the molecular changes following injury (Fig. 2a–d; Supplementary Fig. 4a).

We observed that the neurons distribute in a pattern that reflects dynamics based on their experimental timepoint and connections to naïve or injury states (Fig. 2b–d and Supplementary Fig 4a). In the UMAP visualization, neurons at a naïve (0 day) state are located at the periphery, while neurons post-injury begin to shift toward the center. By 28 days, this phenotype reversed, and most neurons returned to the periphery of the graph (Fig. 2d). To investigate these molecular changes further, we classified the generated Leiden clusters where those spatially distinct areas were classified into “woPain” (without pain) and “wPain” (with pain) macrostates based on their alignment with the “Reference” state (naïve and sham conditions) or not, respectively (Fig. 2c). Differential expression analysis confirmed that the “wPain” category was associated with upregulated genes related to axon regeneration and axon guidance, suggesting a regenerative molecular program following injury, as previously reported^22,23^ (Supplementary Data 4).

Importantly, the “wPain” and “woPain” macrostates could be further broken down into microstates to refine cell progression over time. These microstates, combined with injury timepoints, were named *Reference*, *Moving*, *Pain*, *Recovery,* and *Lasting Pain* (Fig. 2e). The *Reference* state (timepoint 0) represents uninjured cells, naturally within “woPain”. The *Pain* state represents the neurons in “wPain” excluding timepoint 28 days. Cells in “wPain” at 28 days post-injury were labeled as being in the *Lasting Pain* state. Remaining cells at post-injury timepoints but exhibited in “woPain” were further distinguished: the early phase of pain development (up to 1.5 days) as *Moving*, and the later phase (1.5–28 days) as *Recovery*. This categorization was based on the observation that all cells were in the “wPain” macrostate at the 1.5-day timepoint, while “woPain” state cells were seen both before and after. Therefore, cells in “woPain” prior to 1.5 days were assumed to be *Moving* toward *Pain*, while cells after were in *Recovery* from *Pain*.

Importantly, the defined microstates were unbiasedly recovered as pain dynamics states using RNA velocity^24^ (computed from CytoTrace^25^ score) (Fig. 2e and Supplementary Fig. 4b) and PAGA graph analysis^26^ (Supplementary Fig. 4c). We also performed Generalized Perron Cluster-Cluster Analysis (GPCCA) using RNA velocity transition matrix and observed a high degree of alignment when we color-coded the GPCCA results by our own defined states. Note that this analysis was performed for each nociceptor subtype with similar results (Supplementary Fig. 4d). Altogether these results unbiasedly indicate that the nociceptors captured in the atlas dynamically progress between different microstates in chronic pain models.

We therefore extracted the driver genes within nociceptors responsible for the microstates transition and performed cell-cell communication analysis to comprehend the origin of cellular changes centering on the communication between nociceptors, immune cells, and satellite glial cells. For the driver genes within nociceptors, the analysis based on the terminal states revealed that many genes belonged to the “regeneration-associated genes” RAGs^22,23^ (Supplementary Data 5), with dynamic activity along the CytoTrace pseudotime. *Sox11* was identified as the sole transcription factor (TF) that consistently ranked among the top five driver genes in all nociceptor subtypes (Fig. 2f and Supplementary Data 5). We used SCENIC to identify the regulons associated with the molecular programs responsible for controlling the dynamic cell state transition of nociceptors and combined it with an analysis of ATAC peaks’ accessibility after injury (Fig. 2g and Supplementary Fig. 4e, f). Identified regulons included *Atf3*, *Jun*, and *Sox11*. Particularly, *Sox11* regulon activity was found to increase as pseudotime progressed and remained elevated in the *Lasting Pain* state, in contrast to *Atf3* and *Jun* regulons, which exhibited an initial increase in activity followed by a decline (Supplementary Fig. 5a–f). This suggested that SOX11 is a potential driver gene for the development and maintenance of neuropathic pain, a finding that was recently confirmed in vivo^27^, further supporting the robustness of our analysis.

To decipher cell-cell communication patterns (Fig. 2h), we combined all cell types, including non-neuronal cells, and employed tensor decomposition^28^ (Fig. 2h, i; Supplementary Fig. 4g; and Supplementary Data 6). This method extracts multicellular gene expression patterns that vary across different cell types, pain models (contexts), and timepoints to unbiasedly identify groups of molecular ligand-receptor pairs involved in cell-to-cell interactions. In our analysis, these groups, also called “Factors”, capture how molecular changes in nociceptors are related to interactions with immune and satellite glial cells in *Reference* and *non-Reference* (*Moving, Pain, Recovery*, and *Lasting Pain*) states (Supplementary Fig. 4g). Six Factors were revealed, amongst which Factor 3 exhibited the greatest changes between *Reference* and *non-Reference* conditions while affecting all pain models that are included in the iPainDRG (Supplementary Fig. 4g). We, therefore, conducted a gene enrichment analysis using PROGENy pathways on the receptor-ligand pairs characterizing Factor 3 to understand their biological significance. The p53 pathway was the most enriched pathway for this Factor, underlying upregulation of the p53 pathway during pain progression (Fig. 2j). Notably, the distribution of the p53, p21, and p16 tumor suppressor genes within this “Factor” correlated with expression of these genes post-injury conditions in our atlas (Supplementary Fig. 5g). The presence of these genes together strongly suggested a process of cellular senescence during pain chronification^29–31^.

### Development and persistence of pain and senescence of pain-sensing neurons

**Fig. 3:**
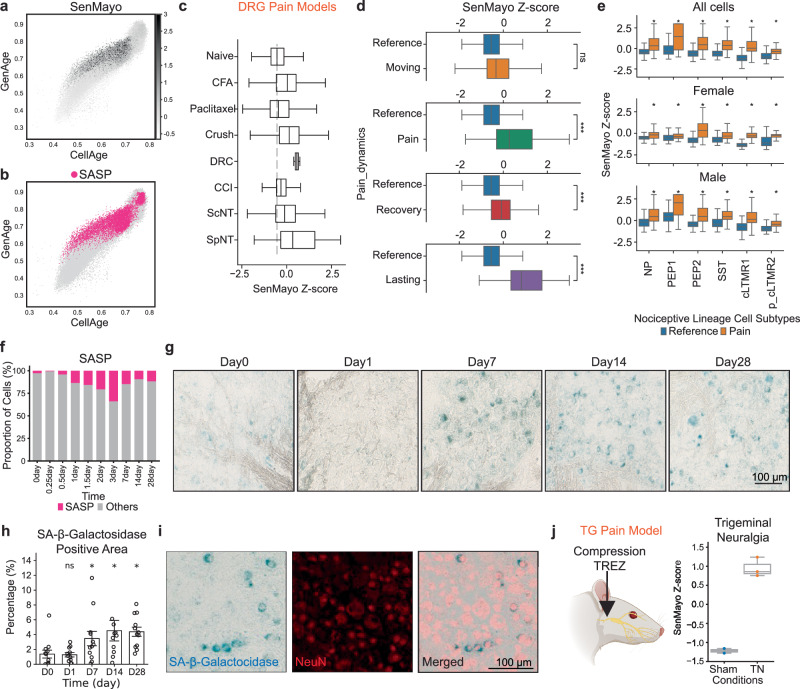
Senescence of nociceptors in pain models in mice with chronic pain. **a**, **b** Scatter plots of nociceptive lineage cells. Cells were plotted based on their CellAge (x-axis) and GenAge (y-axis) scores and colored by SenMayo z-score (**a**) and SASP identity (**b**). **c** Boxplots comparing the SenMayo z-score of nociceptive lineage cells from different neuropathic pain models and control. **d** Boxplots of multiple comparisons comparing SenMayo z-score of nociceptive lineage cells from different states of pain dynamics with the *Reference* state (ns *P* > 0.05; ***P < 0.001, Mann–Whitney U). The statistic was derived from 18,409 cells in *Reference*; 7808 cells in *Moving*; 15,893 cells in *Pain*; 28,199 cells in *Recovery*; and 654 cells in *Lasting*. **e** Boxplots of multiple comparisons comparing SenMayo z-score of different subtypes of the nociceptive lineage cells from *Reference* and *Pain* states. From top to bottom, cells from both sexes together; cells from female mice; and cells from male mice (*P < 0.05, Mann–Whitney U). **f** Bar plot representing the increased proportion of SASP cells as injury time progressed. **g** SA-β-Galactosidase staining images of DRG before and after injury in mice with CCI model, scale bar denotes a length of 100 µm. **h** A bar plot of mean values ± SEM representing the quantification of SA-β-Galactosidase area in injured condition with statistical level to denote the significant level of the positive area in each timepoint after injury when compared to the day 0 (ns *P* > 0.05; *P < 0.05; 0.876, 0.0465, 0.0259, 0.0129 were the *P*-values of D1, D7, D14, and D28 respectively. The test was done with one-sided Mann–Whitney test U). The quantification was derived from 12 DRG samples (L4-L6) of 4 animals at each timepoint, except for D14 where there were 10 DRG samples. **i** Staining images representing the co-staining of SA-β-Galactosidase and NeuN when merged. **j** Left, scheme representing a model of trigeminal neuralgia (TN) in rats, by Trigeminal Entry Zone (TREZ) compression. Right, A boxplot representing the SenMayo z-score between sham and TN conditions in TG tissue RNA-seq data from Tao et al.^37^ (*n* = 2 samples from sham and *n* = 3 samples from TN). **j** Scheme on the left was created with BioRender.com released under a Creative Commons Attribution-NonCommercial-NoDerivs 4.0 International license. Source data are provided as a Source Data file.

The concept of cellular senescence was initially discovered in dividing cells but later extended to include post-mitotic cells, correlating this process with aging in various tissues. Cellular senescence involves cells altering their characteristic phenotype in response to stress, resulting in a distinct secretory phenotype, called the Senescence Associated Secretory Phenotype (SASP), and characterized by the release of inflammatory cytokines, growth factors, and proteases. SASP operates through diverse mechanisms, often involving autocrine or paracrine signaling. To facilitate standardized and streamlined senescence analysis, the Mayo Clinic has recently introduced the SenMayo gene set^32^, encompassing an extensive collection of SASP-related genes (Supplementary Data 7). Using this method, we identified SASP-positive cells by selecting those within the top 10 percent of the SenMayo z-score (Fig. 3a, b) and highlighted them in the scatter plot, depicting GenAge score (gene set associated with aging in model organisms) in relation to CellAge score (gene set associated with aging in human cells)^33^ (Fig. 3a, b and Supplementary Data 8). This analysis demonstrated that cells affected by injury exhibited advancement in both GenAge and CellAge compared to naïve cells.

Upon evaluating this with the CytoTrace score which demonstrated a lower potential for cell plasticity after injury in a subset of nociceptors (Supplementary Fig. 4b), we formulated a hypothesis suggesting that the persistence of pain in the chronic injury model might be attributed to the persistence of injury-induced senescent cells which are unable to reverse their phenotype to the *Reference* state. To test this hypothesis, we initially identified senescent cells among the nociceptive lineage cells by computing a gene module score based on the genes listed in SenMayo. Upon the evaluation of the score, we observed that the SenMayo z-score increased in every type of neuropathic pain model when compared to the control (Fig. 3c). Statistical testing of the SenMayo z-score revealed a high enrichment of senescent cellular characteristics within nociceptors following injury in all pain models except Paclitaxel (Fig. 3c), across the dynamic states (Fig. 3d) and for all nociceptors subtypes, regardless of the biological sexes (Fig. 3e). Additionally, the SenMayo z-score was found to be significantly lower in the *Recovery* state when compared to the *Pain* and *Lasting Pain* states (Fig. 3d). Interestingly, every subtype of the nociceptive lineage cells exhibited the increase of the SenMayo z-score when we compared the *Pain* state to *Reference* state. We then verified the time course of senescence development in silico (Fig. 3f) and in vivo by detecting the Senescence-Associated Beta-galactosidase (SA-β-Gal) staining in DRG after nerve injury (CCI, Fig. 3g, h). We identified a senescent phenotype in a subset of nociceptors, which were characterized by high expression of the neuronal nuclei marker (NeuN) and their smaller size (Fig. 3i). Furthermore, the injury was accompanied by an increased nuclear expression of the senescence marker p21 starting from day 7 post-injury^34^ (Supplementary Fig. 5g, h). These in vivo findings support the reliability of using the SenMayo z-score as a hallmark of senescence in our in silico analysis.

For TG, the atlas covered limited timepoints for proper analysis. We, therefore, assessed the SenMayo z-score in a pain model of compression of the trigeminal root entry zone (TREZ) of the TG nerve, modeling excruciating pain disorders such as trigeminal neuralgia (TN, also called the suicidal disease)^35^, and cluster headache (CH, a trigeminal autonomic cephalalgia) which are both associated with nerve compression by blood vessels (called microvascular compression)^36^. To do this, we used the bulk RNA-seq dataset^37^ from the rat model with TN to compute the score. We identified a clear increase in the SenMayo z-score compared to control conditions suggesting that both the DRG and TG use senescence as a pain chronification mechanism (Fig. 3j).

**Fig. 4:**
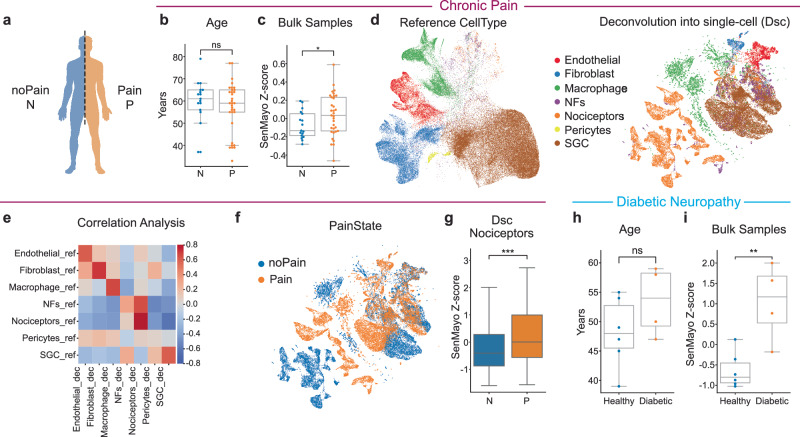
Senescence in humans with chronic pain and diabetic painful neuropathy. **a** A scheme depicting human sample nomenclature (N noPain, P Pain). **b** A boxplot showing that the average age between patients with pain was not significantly different from patients without pain (ns *P* > 0.05, one-sided *t*-test). **c** A boxplot representing the SenMayo z-score with indicated statistical significance for the higher average score in patients with pain (*P = 0.0415, one-sided *t*-test). For **b** and **c** *n* = 17 DRG samples of the noPain (control) cohort and *n* = 33 DRG samples of patients in the Pain group. **d** Left, a UMAP plot of hDRG single-nuclei RNA sequencing from Jung et al. ^39^, cells used to create the reference cells used for deconvolution of bulk into single cells. Right, a UMAP plot of deconvolved cells from hDRG bulk RNA-seq colored by different cell types. **e** A heatmap plot representing the Spearman correlation between the cell types of the reference count matrix (_ref) and the reconstructed count matrix from deconvolution (_decon). **f** A UMAP plot of deconvolved cells from hDRG bulk RNA-seq colored by different known pain conditions. **g** A boxplot comparing the SenMayo z-score from human nociceptive lineage cells of different pain states after bulk deconvolution to single-cell with asterisks to denote the level of significance (***P = 3.545 × 10^−^^91^, one-sided Mann–Whitney U) There were 7418 neurons from control group and 6198 neurons from patients with pain. **h** Boxplot showing that the average ages between patients with diabetic painful neuropathy and the control cohort were not significantly different (ns *P* > 0.05, one-sided *t*-test). **i** Boxplot representing the SenMayo z-score with indicated statistical significance for the higher average score in patients with diabetic pain (*P = 0.00952, one-sided *t*-test). Bonferroni correction was performed to adjust for multiple hypothesis testing on this figure when appropriate with a family-wise error rate of 0.05. Boxplots indicate the median at the center, upper and lower quartiles at the bounds of the box, whiskers are at minima and maxima. Source data are provided as a Source Data file.

We then assessed the SenMayo z-score in human samples focusing on the DRG (TG dataset or samples from pain sufferers are not currently available online nor in biobank). SenMayo z-score was elevated in DRG from human patients diagnosed with chronic pain (Fig. 4a) irrespective of their biological age (Fig. 4b, c), in an online dataset^38^. Deconvolution into cell types using human reference map^39^ (Fig. 4d–f; Supplementary Fig. 5i) showed that the nociceptors (Fig. 4g) have a SenMayo z-score significantly elevated in chronic pain condition, further emphasizing the involvement of senescence in nociceptors in human with chronic pain. Similar results were observed when the SenMayo z-score was applied to DRG bulk-seq^40^ extracted from patients with diabetes neuropathy (Fig. 4h, i). These results in humans support the translational value of our findings. Importantly in mice, the senescence score was significantly higher in all pain models, regardless of the biological sex (Fig. 3c, e).

#### Targeting senescent cells treats chronic pain

While the presence of senescent neurons in peripheral somatosensory neurons was unexpected in the context of chronic pain independent of aging, drugs targeting senescence cells, known as senolytic agents, have been well characterized and are used already in preclinical and clinical trials. Senolytic drugs capitalize on senescent-cell anti-apoptotic pathways (SCAPs)^41^. Disrupting the expression of proteins within these pathways can lead to the selective elimination of senescent cells while preserving neighboring cells and tissue physiology. The senolytic agents used in this study included the bioavailable small molecule inhibitors of B-cell lymphoma-2 protein (Bcl-2) family proteins, such as Navitoclax (ABT-263), Venetoclax, and proteolysis targeting chimera (PROTAC) compound, PROTAC Bcl-XL degrader, the latter aiming at controlling the linked side effects that this class of molecules has on thrombocytes counts.

**Fig. 5:**
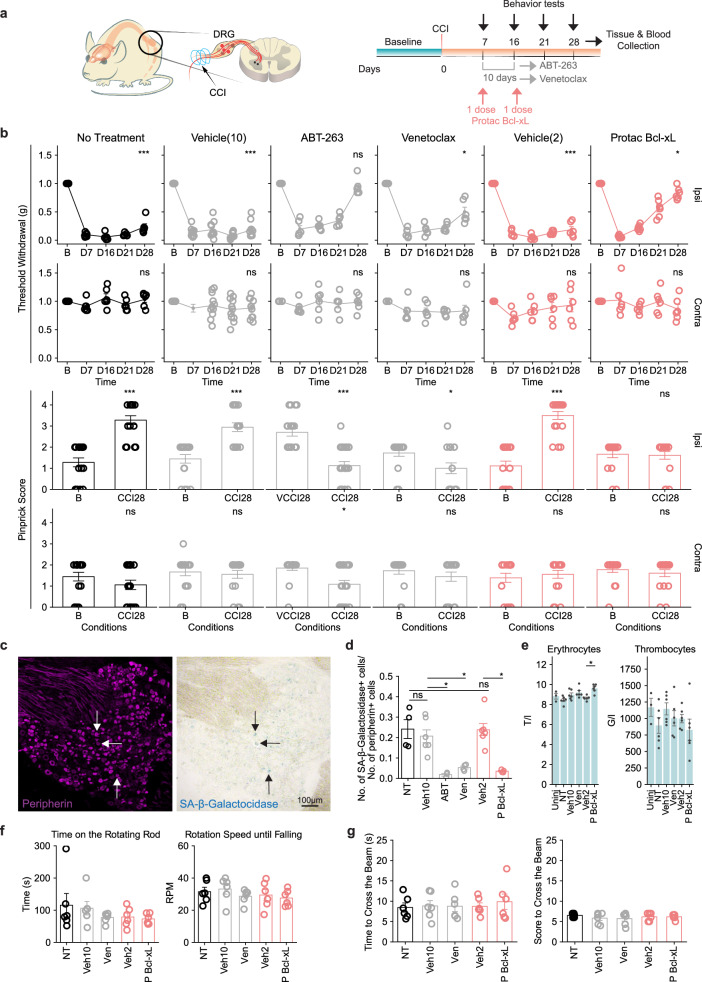
Senolytic compounds provide pain relief in a mouse model of neuropathic pain. **a** Scheme of chronic constriction injury (CCI) pain model and experimental timeline for treatment and behavior tests. **b** Upper, line graphs representing von Frey withdrawal threshold at baseline (“B”) and different timepoints after injury for different treatment conditions on ipsilateral (top panel) and contralateral side (bottom panel) (ns *P* > 0.05; *P < 0.05; ***P < 0.001, two-sided paired *t*-test). Lower, bar plots comparing pinprick score from the same animals at baseline (“B”, before injury) with the pinprick score at different treatment conditions at day 28 after injury (CCI28). Note that for the ABT-263 treatment, the control condition is vehicle 28 days post CCI (“VCCI28”) and (ns *P* > 0.05; *P < 0.05; ***P < 0.001), two-sided paired *t*-test (comparing to “B”) or two-sided unpaired *t*-test (comparing to “VCCI28”). **c** Images of DRG treated with the vehicle at CCI day 28, stained with Peripherin (top) and SA-β-Galactosidase (bottom). Arrows indicate some double-positive neurons for peripherin and SA-β-Galactosidase as an example. **d** Bar plot for staining quantification depicting the proportion of SA-β-Galactosidase positive to peripherin positive cells at CCI day 28 in the different treatment conditions (ns *P* > 0.05; *P < 0.05, one-sided *t*-test; Veh10 and Veh2 were compared with NT; ABT and Ven were compared with Veh10; P Bcl-xL was compared with Veh2). The quantification was derived from DRG (L4) from 4 mice, except the vehicle group where DRG (L4) from 6 mice were used. **e** Bar plots for whole blood count of erythrocytes and thrombocytes for each treatment condition (ns P > 0.05; *P < 0.05, two-sided *t*-test). **f** Bar plots depicting results from the RotaRod test for each treatment condition but ABT-263 (not tested) (ns *P* > 0.05, two-sided *t*-test). **g** Bar plots depicting results from the beam walk test (width 11 mm) for each treatment condition but ABT-263 (not tested) (ns *P* > 0.05, two-sided *t*-test). 6 mice were employed in each group for the behavior test, except the vehicle group where 11 mice were employed. Bonferroni correction was performed to adjust for multiple hypothesis testing when appropriate with a family-wise error rate of 0.05. All bar plots and line graphs represent the mean values ± SEM. Note on abbreviation, Uninj: Uninjured; NT: No Treatment; Veh10: Vehicle (10 doses of DMSO); Veh2: Vehicle (2 doses of DMSO); ABT: ABT-263; Ven: Venetoclax; and P Bcl-xL: Protac Bcl-xL. Source data are provided as a Source Data file.

To test the hypothesis that targeting senescent cells could suppress hypersensitivity to pain in a chronic pain model, we gave these various senolytic agents or vehicles to mice which have been subjected to Chronic Constriction Injury (CCI) and developed hyperalgesia and allodynia by day 7 (Fig. 5a). Remarkably, mice treated with senolytic agents exhibited significantly less or no signs of mechanical pain 4 weeks post nerve injury when compared with baseline or vehicle at the same timepoint (Fig. 5b). The drugs however did not significantly impact thermal sensation during the timeframe of the experiment or at the administered doses. The restoration of normal pain behavior was associated with the selective removal of at least 50% of SA-β-galactosidase positive cells, on average, among the small diameter, peripherin-positives neurons for all drugs (Fig. 5c, d). Ultimately, the removed cells represent a small fraction of the total peripherin-positive neurons. Moreover, body weight remained unchanged (Supplementary Fig. 5j) throughout the experiment and blood counts were similar between the drug-treated and control groups (Fig. 5e), indicating that the senolytic compounds did not show obvious adverse systemic effects and did not significantly affect the thrombocytes counts (Fig. 5e). Importantly, as a control to assess the specific role of the senolytic compounds on pain-sensing neurons and to control for off-target effects, we tested the mice for anxiety balance and motor skills (Fig. 5f, g, and Supplementary Fig. 5k, l). No significant difference was observed when comparing the drug-treated mice to the control conditions. These results provide a more comprehensive understanding of the effects of the senolytic compounds highlighting their selective impact on mechanical hypersensitivity without affecting other sensory modalities or general health parameters.

Together these data indicate that a senescence process occurs in a subset of nociceptive neurons in various chronic pain models and participates in the persistence of the pain state and that targeting these senescent cells represents a promising treatment option for patients affected with chronic pain.

## Discussion

The extensive prevalence of chronic pain in the human population aligns with the pressing need to develop novel approaches to medical treatment. A major limitation to progress in this field has been a lack of comprehensive understanding of the overall phenotypic alterations that underlie the dysfunction of nociceptive neurons following nerve injury, leading to the onset of the chronic pain state. Our study introduces an integrated pain atlas (iPain) for systematically exploring the trajectory of chronic pain development through the analysis of single-cell omics data from various sources. A salient discovery from our research is the consistent induction of a senescence phenotype in the transcriptome of a subset of somatosensory neurons, principally in nociceptors, following peripheral nerve injury, a phenomenon observed in both rodents and human datasets. Pharmacologically targeting senescent cells further proved effective in rescuing pain behaviors in a mouse model of chronic pain, suggesting a role of senescent cells in sustaining a neuropathic pain phenotype. Our data provide functional evidence for the role of senescent cells in the progression and persistence of chronic pain in a sex-independent manner.

This study reveals a dynamic “intrinsic” shift in gene expression patterns in time following peripheral nerve injury. A common transcriptomic signature across all subtypes is *Sox11*. SOX11 is a transcription factor involved in nervous system development and in neurogenesis in adults where it induces the expression of neuronal traits^42,43^. This suggests a role in the regeneration process. However, while the expression of the known regeneration-associated genes like *Atf3* and *Jun* increases at early timepoints post-injury, they decrease later. In contrast, *Sox11* remains elevated at a later timepoint, suggesting a different role than regeneration which would align with persistent pain development and maintenance. Interestingly, in relation to chronic pain a role of *Sox11* was previously predicted using the neuropathic pain model of spared nerve injury^44^, and in a sex-stratified genome-wide association study of Multisite Chronic Pain (MCP), SOX11 was shown to be associated with MCP in females^44^. Finally, it is now reported using loss and gain of function that SOX11 has an important role in the initiation and maintenance of neuropathic pain^25^. Those findings further support the resourcefulness of iPain. Moreover, our approach and our workflow differ from a cross-species atlas^45^, which also integrates data from multiple sources, but focuses on addressing molecular conservation across different species. In contrast, iPain is specifically tailored to charting the trajectory of chronic pain development in a targeted manner.

Previous research has linked cellular senescence with chronic pain, specifically in the spinal cord^46,47^, presumably as a secondary process occurring several months after injury and exhibiting a male-specific effect^46^ in certain cases. Nevertheless, using senolytics to treat the spinal cord locally did not provide sustained relief from neuropathic pain, indicating that this approach did not address the primary mechanism underlying neuropathic pain in the studied model. However, our study found that the connection between cellular senescence and chronic pain begins just after the acute phase, and is linked to the onset of neuropathic/chronic pain. We observed senescence in nociceptors across various models of peripheral nerve injury and inflammation, as well as in the TG for the TREZ compression model. Furthermore, we discovered that this effect is not sex-specific; both male and female animals showed similar levels of cellular senescence in nociceptors. This suggests an underlying mechanism different from those previously reported. Lastly, while our study focuses on young and middle-aged adult animals, this mechanism might also function or even intensify in older individuals in which the development of a senescent phenotype in nociceptors following nerve injury or in control conditions might be more easily induced^48^.

In conclusion, our single-cell transcriptomic atlases could provide substantial advances in drug discovery efforts by defining cells and pathways relevant to pain disorders following peripheral nerve injury. Our discovery suggests an alternative therapeutic approach to the currently limited options for chronic pain treatment, a hypothesis that has been validated in mice with translational potential for human subjects according to in silico analysis of chronic pain patients and patients with diabetes painful neuropathy. The selective removal of the few dysfunctional, senescent neurons may be a worthwhile trade-off to achieve lasting well-being for the patient, especially in the case of excruciating pain conditions with limited options, such as TN and CH which are linked to trigeminal nerve compression^36^.

The current clinical development of several senolytic compounds, including those used in this study, for anti-aging and cancer-related diseases, supports the strategy of targeting senescence to treat persistent pain in humans. This progress suggests these senolytic compounds could soon be repurposed for treating chronic pain and headache disorders resulting from nerve tissue injury. Targeting senescent cells through apoptosis induction is a straightforward method, and there are various agents demonstrating the effectiveness of senolytic agents in vivo, as well as in clinical trials^39^. Therefore, the use of senolytics represents a promising approach in managing chronic pain.

## Methods

The basic analysis of the data was done with the functions from Scanpy^49^ package (v1.9.3) of Python3 (v3.10.10) and default parameters according to Scanpy pipeline unless otherwise specified.

### Mice

Cohorts of adult mice of both biological sexes, aged from 8 to 12 weeks were used for sequencing in all the different published studies^5,9–13^. Our sequencing data on TG and DRG were obtained from mice aged between 8 and 12 weeks using the Smart-seq3Xpress, 10x multiomic technologies, in iPain these mice are referred to as young. A middle-aged cohort (34–62 weeks) was processed using 10x multiomic sequencing, in iPain these mice are referred to as aged. For all details on biological sex, and the number of cells per timepoints see tables in Fig. S2 and the CellxGene^50^ iPain (iPain).

For the senescence kinetic and for the drug treatment experiment, cohorts of mice aged between 17 and 27 weeks of both sexes (3 males and 3 females per group) were used.

All the mice were on a C57BL/6 background and were kept under a 12-h light–dark cycle, at 24 °C with unlimited food and water. All animal care and experimental procedures were permitted by the Ethical Committee on Animal Experiments (Stockholm North committee) and conducted according to The Swedish Animal Agency’s Provisions and Guidelines for Animals Experimentation recommendations. Ethical permit numbers 9702-2018 and 17396-2022.

### Drug administration

The senolytic compounds were given orally, by gavage. The stock solution was diluted as 10% DMSO in corn oil prior to administration. The treatment starts seven days post CCI injury, the senolytic compound or vehicle control (10% DMSO in corn oil) was administered by oral gavage once daily for either 10 consecutive days (Navitoclax CAS No. CAS. 923564-51-6 or Venetoclax CAS No. 1257044-40-8) or once weekly for 2 weeks PROTAC Bcl-xL degrader (CAS No. 2920415-08-1).

### Full blood Count

Mice were deeply anesthetized with isoflurane. The full blood was taken from the hearts of the mice and kept in a blood collection tube (containing EDTA) for further testing by IDEXX BioAnalytics.

### Chronic constrictive injury mice model

Chronic constriction injury of the sciatic nerve (CCI) has been widely described with minor differences in rodents^51–53^. Briefly, mice were first anesthetized with isoflurane, and a small incision was made at the mid-thigh level on the right side. Ligatures (7–0 surgical silk) were tied loosely around the sciatic nerve with approximately 0.5 mm between ligatures. And then, the skin was closed by a 5–0 silk suture.

### Pain-related behavioral tests

Mechanical sensitivity was assessed by the von Frey test in an up-down testing paradigm as previously described^54,55^. Briefly, mice were placed in glass cylinders on a 6 × 6 mm wire mesh grid floor and were allowed to acclimate for 20–60 min. The plantar surface of the hind paw was stimulated with a series of calibrated monofilaments (von Frey hairs; Stoelting, IL) ranging between 0.008 and 2 g and withdrawal or jerking of the paw is recorded as the pain threshold. Mice were tested 6 times on each paw following the testing paradigm.

Thermal sensitivity was assessed by the Hargreaves thermal paw withdrawal test as previously described^56,57^. Briefly, mice were placed in Plexiglas chambers on top of a glass sheet and were allowed to acclimate for 20–60 min. A thermal stimulator (IITC Inc., Woodland Hills, CA, United States) was precisely aimed at the middle of the plantar surface of the mice through the glass sheet. The laser intensity was set to 30% and a cutoff latency of 20 s was set to avoid tissue damage.

Noxious mechanical sensitivity was assessed by pinprick test as previously described with minor modification^58^. Briefly, mice were placed in Plexiglas chambers on top of a glass sheet and were allowed to acclimate for 20–60 min. A 25-gauge needle connected with a 1 g filament (von Frey hairs; Stoelting, IL) was applied uniformly to the plantar surface of the hind paw without penetrating the skin. A score system was used according to the extent of the response. 0 = no response; 1 = move, look around to see what happened; 2 = brief quick lift or withdrawal or removal away of hind paw; 3 = brief quick shakes of the hind paw, or jumps; 4 = high frequency of shaking, licking, flinching, or guarding. Mice were tested 4 times on each paw with a waiting time of 5min^58,59^.

### Anxiety and balance motor function-related behavior assessments

Anxiety was assessed by the open field test (OFT) and elevated plus maze (EPM) tests^60,61^. For OFT, animals were placed in a roofless 45 × 45 cm square arena enclosed with 40 cm walls and allowed to explore for 15 min while being recorded by a camera secured on the ceiling. Tracking and analysis were done using the EthoVision XT software where the arena was further partitioned into two zones: center (square area of 22.5 × 22.5 cm in the middle of the arena) and periphery (rest of arena close to walls). The proportion of time spent in the two different zones with respect to the center-point of each animal was used as an indicator of anxiety.

For EPM, animals were placed in a plus (+) shaped arena elevated at 60 cm from the ground and allowed to explore for 5 min while being recorded by a camera secured on the ceiling. Tracking and analysis were done using the EthoVision XT software where the arena was partitioned according to its design: two closed arms opposite to each other that are each 6 × 35 cm enclosed by 15 cm walls, two open arms opposite to each other with 6 × 35 cm which are all connected to one another by a small 6x6cm center area. The total distance traveled, and the proportion of time spent in the different types of zones with respect to the center-point of each animal were used as indicators of anxiety.

Motor coordination was assessed by beam walk and rotarod tests^62–65^. For the beam walk test, each animal and some bedding material was first placed for 2 min for habituation inside the goal box, which is 12 × 12 × 12 cm elevated to 64 cm from the ground and enclosed on all sides except for the entry side. A round beam with a diameter of 11 mm (Small) and a length of 105 cm were used. The test beam was placed straight with the endpoint in front of the goal box parallel to the ground with start and finish lines defined for a total length of 80 cm in the middle of the beam. Animals were first habituated on a 20 mm beam until the ability to fully cross the beam without hesitation was demonstrated. A total of three trials were performed on the 11 mm beam, with at least 1 min rest between trials in the goal box. Each trial was recorded on camera and the time required for the mouse to cross the beam from start to finish and the usage of the injured leg as a scoring system were used as indicators of motor function. 7 = traverses beam normally with no more than two footslips; 6 = traverses beam successfully and uses affected limb in more than 50% of the steps; 5 = traverses beam successfully and uses affected limb in less than 50% of the steps; 4 = traverses beam and places affected limb on horizontal surface at least once; 3 = traverses beam by dragging affected limbs; 2 = the animals cannot traverse but were able to place limbs on horizontal surface and maintain balance; 1 = the animal cannot traverse and were unable to put affected limb on horizontal surface.

For rotarod, the equipment from Ugo Basile S.R.L. model 47,600 was used with a start speed of 4 rpm/min, a max speed of 40 rpm, and a ramp speed of 1.8 min (20 rpm/min). Mice were placed on the rotating dowel set to minimum speed and allowed to habituate for 5 min on the first trial. A total of 8 trials were performed for each animal with a rest time of at least 5 min between each trial in the home cage. The time and final rpm of the mouse at falling or the first occurrence of passive rotation (clinging to the dowel) were used as indicators of motor function. The first 2 trials were seen as habituation trials and the last 5 trials were taken for analysis.

### Senescence-associated β-galactosidase staining

Mice were deeply euthanized with isoflurane and perfused with pre-cooled PBS. L4- L6 DRGs were quickly dissected, immediately frozen, and then quickly imbedded in optimal cutting temperature embedding medium (OCT). The SA-β-galactosidase activity was detected by using a SA-β-galactosidase staining kit (Cell Signaling Technology, #9860) at PH 6.0 according to the manufacturer’s instructions with minor modification. Briefly, DRG sections were rinsed twice by PBS before fixation, followed by x-gal (PH 6.0) staining. 4 days after incubation (at 37 °C), the staining solution was removed, rinsed three times with PBS, sealed, and then immediately taken imaging.

### Immunostaining

The snap-frozen DRG tissues were collected as described above (in “Methods” section, SA-β-galactosidase staining section). The snap-frozen DRG tissue was either freshly sectioned and then fixed in PFA overnight at 4 °C, or reused from SA-β-galactosidase staining (double staining). DRG sections were washed with PBS, and permeabilized with 0.3% PBST for 10 min, followed by incubation in blocking buffer (PBS containing 0.25% Triton X-100 and 1% bovine serum albumin) for 30 min at room temperature (RT). And then, DRG sections were incubated with primary antibody (diluted in blocking buffer) at 4 °C overnight. After 3 washes with blocking buffer, a secondary antibody (1:1000, diluted in blocking buffer) was applied for 1 h at RT. Cells were then incubated in DAPI solution (D212, Wako) for another 10 min, followed by 3 washes with PBS. Images were obtained with a fluorescence microscope or confocal microscope.

The following primary antibodies were used: anti-p21 antibody (1:100, gift from Doctor Sylvain Peuget), anti-peripherin (1:200; ab39374, Abcam), anti-NeuN (1:200; MAB377, Merck Millipore).

### Imaging

Brightfield images were acquired using the Zeiss Axio Imager.Z2. Images were analyzed with ImageJ (v1.52 h) by (1) manual segmentation of ganglion while excluding large fibers; (2) general isolation of the blue stain with the Color Deconvolution plugin^66,67^ with the built-in Brilliant Blue vector; (3) thresholding by pixel intensity from 5 to 185 with Li’s method and (4) positive stain identification by the Analyze Particles function (size inclusion from 400-Infinity). Identified positive regions were refined manually before measurement of the area which were summed and normalized against the total area of the segmented DRG to determine the percentage positive area for each DRG. Statistical significance was calculated with the Mann–Whitney U test.

### DRG dissociation and single-nuclei isolation^51–53^

Mice were deeply euthanized by isoflurane and perfused with 4% pre-cooled PBS. L4-L6 DRGs were collected at 14 days after CCI injury in aged mice or from young mice (without injury), and then immediately put on dry ice. Frozen tissues are stored at −80 °C before nuclei isolation.

Isolation of nuclei from frozen tissue was performed as described by the Allen Institute for Brain Science^68,69^. All steps were performed in 4 °C. Briefly, tissues were homogenized in 2 ml chilled homogenization buffer (10 mM Tris pH 8, 250 mM Sucrose, 25 mM KCl, 5 mM MgCl2, 0.1 mM DTT, 1× Protease inhibitor cocktail, 0.2 U/μl RNasin Plus, 0.1% Triton X-100), and filtered through 70 µm and 30 µm cell strainers. Nuclei pellets were collected by 10 min, 900 × *g* suspension, and then resuspended in 250 μl chilled homogenization buffer. Finally, debris was removed by Iodixanol 25–29%, and the nuclei pellet was resuspended. For single-nuclei Multiomic sequencing, the nuclei pellet was resuspended in 30 µL 1× chilled nuclei buffer (10x Genomics, 2000207) and then proceeded immediately for single-nuclei Multiomic sequencing protocol. For neuron-specific- single-nuclei RNA sequencing, the nuclei pellet was resuspended in 500 ul blocking buffer (1× PBS, 1% BSA, 0.2 U/μl RNase inhibitor) for fluorescent-activated nuclei sorting and Smart-seq3xpress.

### Fluorescent-activated nuclei sorting and single-nuclei RNA sequencing (Smart-seq3xpress and 10x Multiomic sequencing)

To enable sorting of nuclei derived from neurons, nuclei suspension was incubated with anti-NeuN PE-conjugated antibody (1:500, FCMAB317PE, Merck) for 30 min on ice. Next, nuclei pellet was collected by 5 min, 400 × *g* suspension, and resuspended in PBS containing 0.1 μg/mL DAPI (D3571, Invitrogen) and 0.2 U/ul RNase inhibitor. Single NeuN+ and DAPI+ nuclei were sorted into each well of a 384-well plate containing 0.3 µL lysis reaction mix by a flow cytometer (DB FACSAria Fusion or BD FACSAria III) at 4 °C. Gating was performed based on DAPI and phycoerythrin signal of NeuN. After sorting, each plate was immediately spun down and stored at −80 °C before single-nuclei RNA sequencing with Smart-seq3xpress. The smart-seq3xpress protocol was performed by Rickard Sandberg’s Group, Karolinska Institute. Nuclei isolated for single nuclei Multiomic sequencing were stained with DAPI and sent for sequencing by Eukaryotic Single Cell Genomics Facility at SciLifeLab, Stockholm.

### Obtaining and preprocessing the publicly available scRNA-seq data

The count matrices and associated metadata of DRG were obtained from the Gene Expression Omnibus (GEO) with the following accession numbers presented on Table 1. Additionally, the data were preprocessed according to the original papers before being loaded into Python3 as AnnData objects.

### Preprocessing the lab-generated data

#### DRG 10x multiomics

The samples were sequenced on NovaSeq6000 (NovaSeq Control Software 1.7.5/RTA v3.4.4) with a 50nt(Read1)−8nt(Index1)−24nt(Index2)−49nt(Read2) (ATAC part) and 28nt(Read1)−10nt(Index1)−10nt(Index2)−90nt(Read2) (GEX part) setup using ‘NovaSeqStandard’ workflow in ‘S2’ mode flowcell. The Bcl to FastQ conversion was performed using bcl2fastq_v2.20.0.422 from the CASAVA software suite. The quality scale used is Sanger/phred33/Illumina 1.8+. The sequenced reads of each sample were aligned using Cell Ranger Arc pipeline version 2.0.2 to the mouse genome (mm10). After the alignment, the chromatin peaks from each sample were aggregated together to obtain a common peak set with “aggr” function from Cell Ranger Arc. The count matrices were loaded into Python3 as AnnData objects. The quality control was done by removing features that were detected in less than 3 cells, and cells with a the natural log pseudo count (log1p) of the number of genes or peaks less than 4 and more than 9 were removed. Cells with percent mitochondrial genes of more than 1.5 and a total UMI count of more than 20,000 were also filtered out.

#### Smart-seq3 DRG & TG

The sequenced reads were aligned using the zUMIs pipeline which makes use of the STAR-SOLO^70^ alignment tool, and the reference genome was mouse (mm39). After alignment, the count matrices were loaded into Python3 for analysis. Furthermore, the count matrices were filtered to only yield high-quality cells. For DRG, genes found in less than 20 cells, and cells with less than 1000 genes were filtered out. For TG, genes detected in less than 3 cells, and cells with more than 3 percent of mitochondrial genes and less than 2000 detected genes were removed.

### Main UMAP of DRG

The AnnData objects were concatenated to create a unified AnnData object, and we made use of two models which were based on the scVI^71^ framework (v0.20.3) for the integration of DRG data. First, we trained the scVI model to integrate the DRG cells unbiasedly on 3000 highly variable genes. The model architecture was designed to obtain 50 dimensions in the latent space with 5 hidden layers and 128 nodes per hidden layer, where the distribution of gene likelihood was negative binomial. Then we constructed a scANVI model based on the trained scVI model to obtain the latent space that could clearly separate the respective cell types for visualization. We used the cell-type nomenclature from Renthal et al. ^5^ to train the model and perform label transfer. The scANVI model was trained until the model was converged. After the models were trained, the latent spaces from scVI and scANVI were extracted. The neighbor graph was built from 50 dimensions with 100 neighbors and the method to calculate the distance was cosine based on the latent space from scANVI. The projection of UMAP of constructed from the neighbor graph with parameter min_dist = 0.5 before plotting.

### Feature imputation

Next, we train the MultiVI model to imputed for the missing features across data modalities by using our lab-generated multiomics data as an anchor. We aimed to impute as many features as possible. The MultiVI was constructed by using the default parameters and was trained until it converged. To train the model, we employed NVIDIA A100 GPU to be able to hold the count matrix on the VRAM, and this resource was provided by the National Academic Infrastructure for Supercomputing in Sweden (NAISS). After the training, the imputation of the gene expression modality was done by calling get_normalized_expression method from the model, while the chromatin accessibility modality was imputed using get_accessibility_estimates method with custom parameters of threshold = 0.1, normalize_cells = True, and normalize_regions = True. The imputed matrices were then multiplied by 10,000.

### Analysis of the nociceptive lineage cells

The nociceptive lineage cells were selected and subset to yield high-resolution clusters. The neighbor graph and UMAP projection were calculated using the same parameters as mentioned above. Then we clustered the nociceptive lineage cells with the Leiden clustering algorithm at 0.1 resolution. To visualize the distribution of cells along the time kinetic after injury, the embedding_density method from the Scanpy package was utilized to compute the cell density on the UMAP projection for cells at different timepoints. Next, we ran the pySCENIC^72^ pipeline to calculate the regulon activity in the nociceptive lineage. Yet, not all TFs had the information for regulons and thus we fit the univariate linear model (ULM) from the decoupleR^73^ package by using the RNA expression of each cell as an independent variable and the interaction weight of each TF from the adjacency table from pySCENIC as a target. The t-value of the slope of the fitted model represented the TF activity.

### Cell state potency

The relative cell state potency of the nociceptive lineage cells was calculated in R using the CytoTrace package. Before running CytoTrace, we reconstructed the raw count matrix of the gene expression data from the imputed normalized count with the PyTorch distribution module for the negative binomial distribution. After the reconstruction, the AnnData object was converted into a SingleCellExperiment (SCE) object using rpy2 and anndata2ri Python packages. Once converted, we ran CytoTrace on the reconstructed raw count matrix with data sources passed to the batch parameter. The CytoTrace was normalized to have the range from 0 to 1. Then we calculated the CytoTrace pseudotime by taking the difference between 1 and the normalized CytoTrace score. We performed an independent *t*-test with 7000 random permutations on the CytoTrace scores between the naïve and injury states with the SciPy package to confirm that the score in the naïve state was significantly higher than that of the injured state.

### Temporal analysis of nociceptive subtypes

We employed CellRank^74^ (v2.0.0) to perform the temporal analysis of each nociceptive subtype between control and pain states based on RNA modality. For each subtype, we made use of the CytroTrace kernel from CellRank to compute the transition matrix. Then we passed the transition matrix CytroTrace kernel to Generalized Perron Cluster-Cluster Analysis (GPCCA) for the identification of terminal states and fate probability toward the terminal states with n_states=3. With GPCCA, we identified the lineage driver genes that drove the transition of cells toward the terminal state of pain. A generalized additive model was utilized to fit the expression profile for each gene as a function of pseudotime to visualize the expression trend of the lineage driver genes. Furthermore, we also applied the same analytical approach on the regulons level calculated from SCENIC.

### Cell-cell communication

The inference of cell-cell communication (CCC) was done with the LIANA+^75^ package (v0.1.8). Here, we passed the count matrix of the atlas as input to the rank_aggregate.by_samplen function of LIANA and we selected “mouseconsensus” as the resource for CCC inference. Due to multi-conditions and multi-timepoints information held within the atlas, the communications were very complex. Thus, tensor decomposition was employed to extract meaningful communications as factors. This was accomplished by calling run_tensor_cell2cell_pipeline function provided by the cell2cell^28^ package.

### Identification of senescence

The SenMayo gene score was calculated based on the scaled data of the imputed gene expression count matrix by using the score_genes function from the Scanpy package. The SenMayo gene set can be found in Supplementary Data 7. Additionally, we selected cells within the top 10 percentile and assigned them as SASP. To study the relative age of SASP cells, we performed gene set enrichment analysis with the run_gsea function from the decoupleR^73^ package to extract the enrichment scores of GenAge and CellAge gene sets. This was done on the normalized unscaled imputed gene expression count matrix. Mann–Whitney U test was performed to test for the statistical significance of the SenMayo score between the naïve and injury groups.

### Analysis of human DRG RNA-seq within

For the analysis of human DRG RNA-seq, we first obtained the bulk RNA-seq data of different pain conditions of neuron-rich samples from Ray et al.^38^. As the count matrix was normalized using the TPM method, we multiplied 1e^6^ to the matrix to unnormalize it back to the raw transcript count values. Then we normalized the raw count to obtain the total transcript count per sample after normalization equal to the median of the total transcript count for that sample before normalization. The normalized count matrix was log-transformed and scaled with log1p and scale functions from Scanpy. SenMayo score was calculated with the score_gene function on the scaled count matrix.

Regarding the deconvolution of hDRG, we retrieved the snRNA-seq of hDRG under normal conditions from Jung et al.^39^ with the accession number GSE201654 from GEO. The data was preprocessed and analyzed as described in the original paper. The deconvolution was done using a VAE-based model called Bulk2Single provided by the omicverse^76^ package in Python3. The bulk RNA-seq data of hDRG was separated into two count matrices, one from donors without known pain conditions and the other from donors with pain. As the data was split in two, we trained two models separately for 3500 epochs each. After the training, the predicted snRNA-seq count matrices were reconstructed and merged for further analysis. There were more than 250,000 cells generated by the models in total. We processed and clustered the matrix according to Scanpy workflow with the Leiden clustering algorithm, and we noticed that there was a lot of noise within the generated data as there were many clusters comprised of 50–1000 cells. Thus, Leiden clusters with less than 900 cells were removed to retain cells with clean signals. Furthermore, we selected only nociceptive cells to calculate the SenMayo score after scaling the count matrix.

Regarding the analysis of hDRG, we noted that the cell proportions between the reference snRNA-seq and the deconvolution of bulk RNA-seq were not equal. This result was expected as we performed the deconvolution on the samples from the group being classified by the authors as neuron-rich samples^36^ (51 out of 70 samples), to focus our analysis on nociceptive lineage cells. The deconvolution yielded ~60% neurons whereas the reference snRNA-seq contained a more representative population of the cell types in DRG with around ~2% neurons. We thus applied a broad classification on the hDRG in contrast to the single-cell analysis in mice model, as we suspected that the deconvolution model could not perform well in classifying the high-resolution cell types (neuronal subtypes) that were closely related to each other due to it being trained on the reference sample with lower neuron representation. Nevertheless, even with the broad classification the model seems to have some difficulties in clearly distinguishing between the two neuronal cell types of NFs and nociceptors that are more highly correlated than other cell types (Fig. 4h). Despite this, the Spearman correlation still indicated the highest correlation between the transcriptome profiles of the nociceptors identified in the reference snRNA-seq and the deconvolved RNA-seq nociceptors, indicating the applicability of the model. We then proceeded to perform a SenMayo score comparison between the NoPain and Pain groups to find that it is increased in the Pain group indicating for cellular senescence, aligning with our other results.

### Analysis of human DRG RNA-seq with diabetic neuropathy

The count matrix was obtained from Hall et al.^40^. The count matrix was normalized into transcript per million and pseudo-log transformed. The effect of different sexes was regressed out with Scanpy’s regress_out function. Samples with their ages being outliers between the healthy and diabetic groups were removed; these were samples with ages below 30 and ages above 60. The SenMayo score was calculated after the matrix was being scaled. The Z-score of SenMayo was computed before performing a one-sided *t*-test to statistically compare the scores between healthy and diabetic groups.

### UMAP of TG

For the TG, we generate an integrated atlas separately for TG using scGLUE^20^ (v0.3.2), by first computing the latent space representation of each source and modality and passing them as input. For the latent space representation of RNA, we updated the weights of the scANVI model trained on the DRG data to obtain the updated latent space of the RNA gene expression modality for the TG datasets. LSA was performed on the ATAC modality to obtain the latent semantic indexing from the TG datasets. Furthermore, scGLUE required an additional input besides the latent space representations which was the guidance graph. The guidance graph was constructed using the highly variable features from RNA and ATAC as described in the original paper^20^. After the inputs were prepared, the model was trained with the default parameters. Once the model was trained, we extracted cell embedding from the model to build a neighbor graph using “cosine” as a metric. The UMAP was computed from the neighbor graph. To facilitate us with cell-type annotation, we perform label transfer using the scANVI model that was previously updated with the weights.

### Statistical analysis

The statistical analysis and tests were done in Python3 with the “stats” module from the SciPy v1.10.1 package. The data were either imported as DataFrame or Array through Pandas v2.0.3 or Numpy v1.22.4 package respectively. In general, a *t*-test was applied when the data followed a normal distribution. Otherwise, the Mann–Whitney U test was performed if the data did not appear to follow the normal distribution. For the behavior analysis with paired data paired *t*-test was used for comparison, where an independent *t*-test was used on unpaired data.

### Reporting summary

Further information on research design is available in the Nature Portfolio Reporting Summary linked to this article.

## Supplementary information

Supplementary Information

Peer Review File

Description of Additional Supplementary Files

Supplementary Data 1

Supplementary Data 2

Supplementary Data 3

Supplementary Data 4

Supplementary Data 5

Supplementary Data 6

Supplementary Data 7

Supplementary Data 8

Reporting Summary

## Source data

Source Data

## Data Availability

The sequencing data and count matrices generated from this study have been deposited in the Gene Expression Omnibus (GEO) database under accession code GSE253345. All the datasets with their GEO accessions used to generate iPain can be found in Table 1. The interactive visualization of the atlases can be accessed through CELLxGENE platform from the Chan Zuckerberg Initiative (iPain) and UCSC cell browser^77^. Source data are provided with this paper.

## Code availability

The script for the core analysis of this work can be found at https://github.com/PrachTecha/Hadjab_iPain.
