# Supplementary material for: The single-cell transcriptomic atlas iPain identifies senescence of nociceptors as a therapeutical target for chronic pain treatment
Source: Nat Commun. 2024 Oct 4;15:8585. doi: 10.1038/s41467-024-52052-8 (PMC11450014; doi:10.1038/s41467-024-52052-8)
Supplement: Supplementary file 1 — Supplementary Information [file 41467_2024_52052_MOESM1_ESM.pdf]

Supplementary Figures

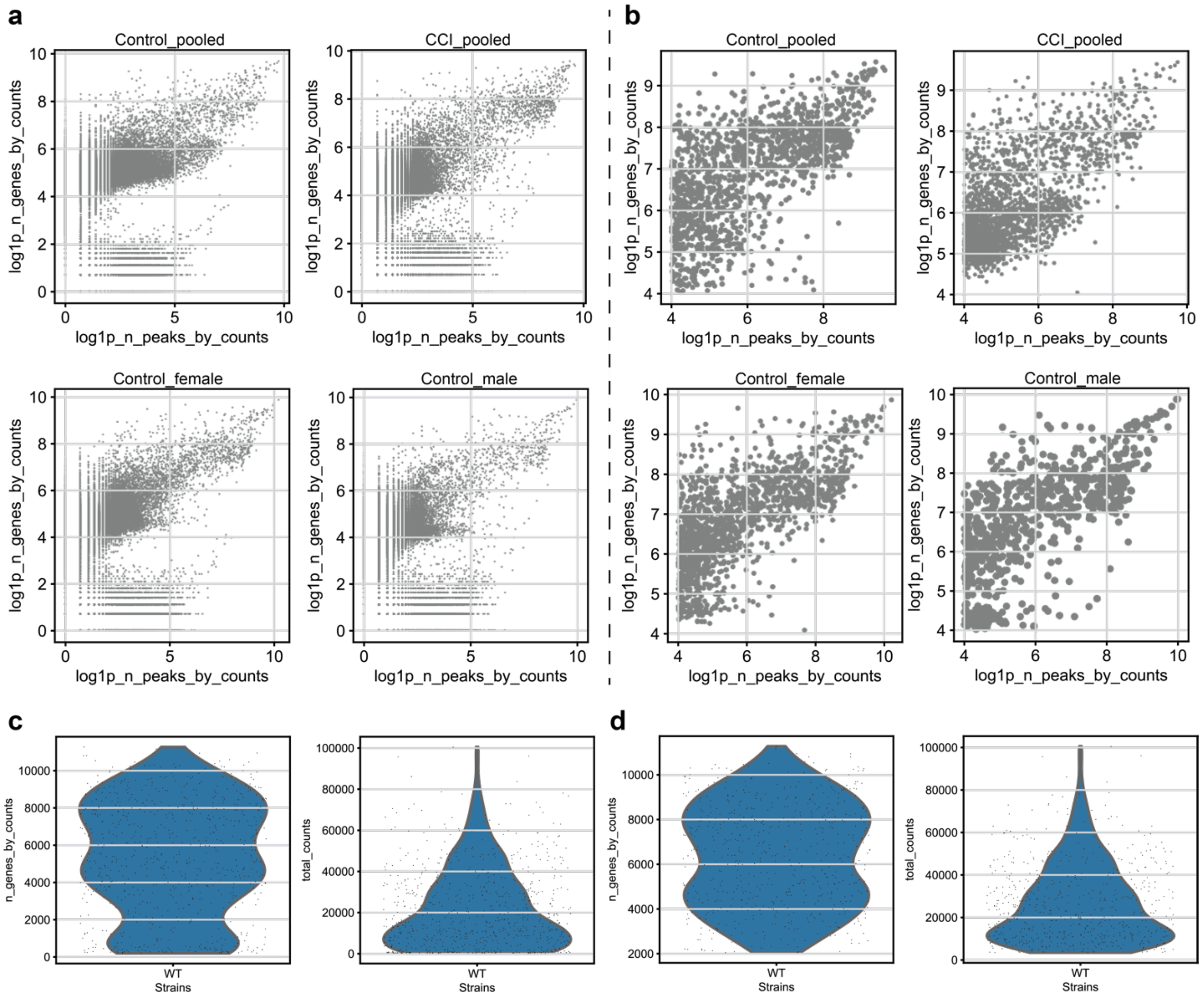

Supplementary Fig. 1: Quality control matrices of DRG and TG data and integration workflow for TG and DRG.

a Scatter plots of the quality control matrices for the in-house generated sn-multiomics data before filtering. **b** Scatter plots after filtering was done on the datasets. **c-d** Violin plot of cells from the generated smartseq3xpress data before the filtering (**c**) and after the filtering (**d**).

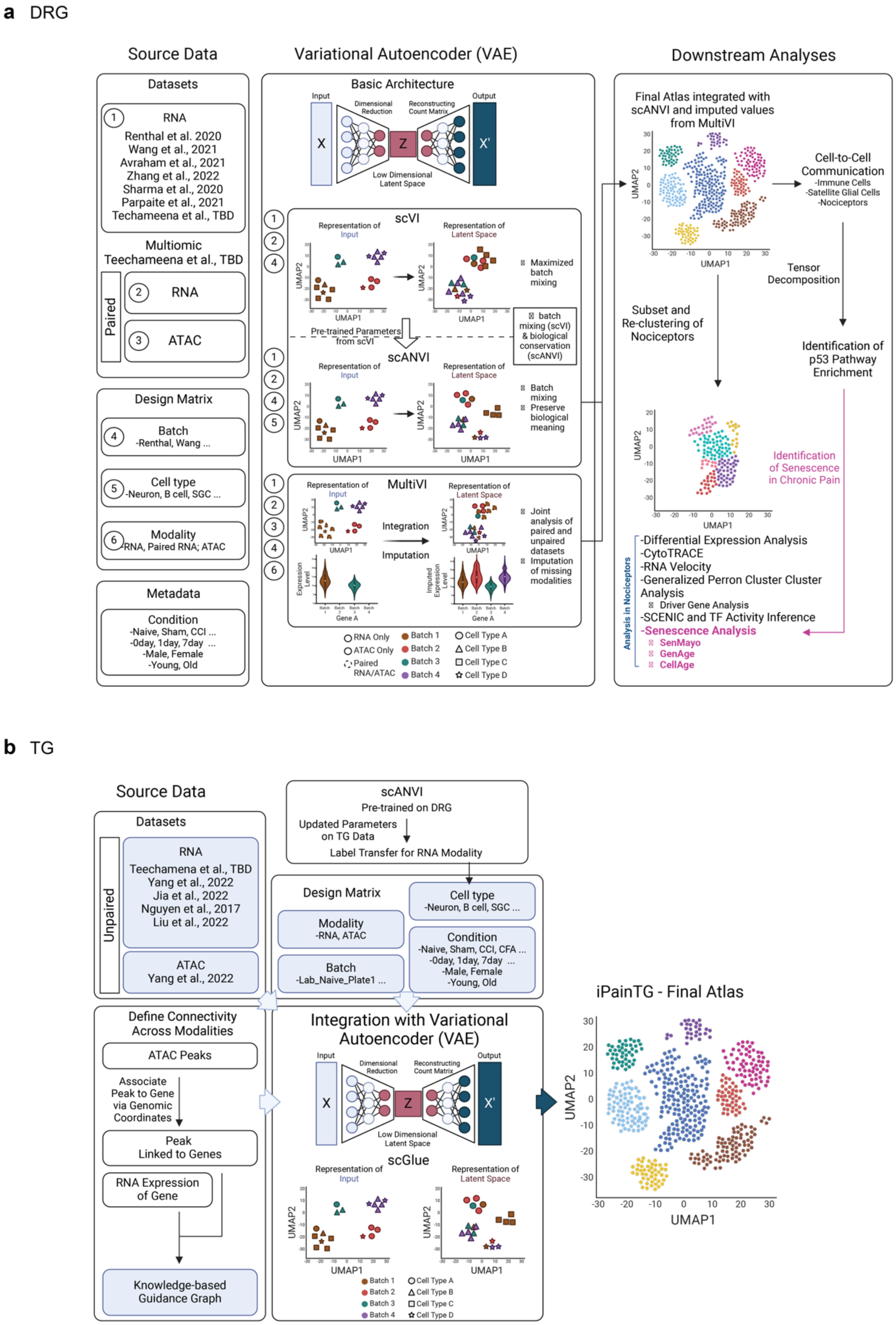
a DRG
Source Data
Variational Autoencoder (VAE)
Downstream Analyses
Datasets
RNA
Renthal et al. 2020
Wang et al., 2021
Avraham et al., 2021
Zhang et al., 2022
Sharma et al., 2020
Parpaite et al., 2021
Techameena et al., TBD
Multiomic
Teechameena et al., TBD
Paired
RNA
ATAC
Design Matrix
Batch
-Renthal, Wang ...
Cell type
-Neuron, B cell, SGC ...
Modality
-RNA, Paired RNA; ATAC
Metadata
Condition
-Naive, Sham, CCI ...
-0day, 1day, 7day ...
-Male, Female
-Young, Old
Basic Architecture
Input
Dimensional Reduction
Reconstructing Count Matrix
Output
X
Z
X'
Low Dimensional Latent Space
scVI
Representation of Input
Representation of Latent Space
UMAP1
UMAP2
Maximized batch mixing
Pre-trained Parameters from scVI
batch mixing (scVI) & biological conservation (scANVI)
scANVI
Batch mixing
Preserve biological meaning
MultiVI
Integration
Imputation
Joint analysis of paired and unpaired datasets
Imputation of missing modalities
Expression Level
Imputed Expression Level
Gene A
RNA Only
ATAC Only
Paired RNA/ATAC
Batch 1
Batch 2
Batch 3
Batch 4
Cell Type A
Cell Type B
Cell Type C
Cell Type D
Final Atlas integrated with scANVI and imputed values from MultiVI
Cell-to-Cell Communication
-Immune Cells
-Satellite Glial Cells
-Nociceptors
Tensor Decomposition
Subset and Re-clustering of Nociceptors
Identification of p53 Pathway Enrichment
Identification of Senescence in Chronic Pain
Analysis in Nociceptors
-Differential Expression Analysis
-CytoTRACE
-RNA Velocity
-Generalized Perron Cluster Cluster Analysis
Driver Gene Analysis
-SCENIC and TF Activity Inference
-Senescence Analysis
SenMayo
GenAge
CellAge
b TG
Source Data
Datasets
Unpaired
RNA
Teechamena et al., TBD
Yang et al., 2022
Jia et al., 2022
Nguyen et al., 2017
Liu et al., 2022
ATAC
Yang et al., 2022
scANVI
Pre-trained on DRG
Updated Parameters on TG Data
Label Transfer for RNA Modality
Design Matrix
Cell type
-Neuron, B cell, SGC ...
Modality
-RNA, ATAC
Condition
-Naive, Sham, CCI, CFA ...
-0day, 1day, 7day ...
-Male, Female
-Young, Old
Batch
-Lab_Naive_Plate1 ...
Define Connectivity Across Modalities
ATAC Peaks
Associate Peak to Gene via Genomic Coordinates
Peak Linked to Genes
RNA Expression of Gene
Knowledge-based Guidance Graph
Integration with Variational Autoencoder (VAE)
scGlue
iPainTG - Final Atlas
UMAP1
UMAP2

Supplementary Fig. 2: The integration workflow for TG and DRG.

a A schematic integration workflow for DRG datasets. **b** A schematic integration workflow for TG datasets. Panel **a** and **b** were created with BioRender.com released under a Creative Commons Attribution-NonCommercial-NoDerivs 4.0 International license.

a DRG: All cells

Conditions	Source	Sex	Control	0.25d	0.5d	1d	1.5d	2d	3d	7d	14d	28d	60d	90d
CFA	Renthal	male	12271*					1495						
Paclitaxel	Renthal	male	12271*							2928				
Crush	Avraham	female	4779*						3358					
	Renthal	female	2781							3167				
		male	12271*	3456		4232			10537	12651	10241	11220	8436	3943
DRC	Avraham	female	4779*						5173					
CCI	Techameena_Multiome	female	1948								1848			
		male	602								70			
	Zhang	female	6634†							4088				
		male	4067†							3251				
ScNT	Renthal	male	12271*	1042	2719	800			1931	4971		2091	1933	
	Wang	male	5333		3104	2735		1981		3535	1657	1306		
SpNT	Avraham	female	4779*						5971					
	Renthal	male	12271*	2201	2431	3024	2277	2610	2413	1201				
Exrtra control	Techameena_SS3	female	371											
		male	254											
	Parpaite	male	49											
	Sharma	female	11132											

DRG: Nociceptors

Conditions	Source	Sex	Control	0.25d	0.5d	1d	1.5d	2d	3d	7d	14d	28d	60d	90d
CFA	Renthal	male	6328*					764						
Paclitaxel	Renthal	male	6328*							1898				
Crush	Avraham	female	6*						11					
	Renthal	female	1441							1781				
		male	6328*	1311		1431			2471	4384	2302	3297	3179	1315
DRC	Avraham	female	6*						3					
CCI	Techameena_Multiome	female	311								368			
		male	125								17			
	Zhang	female	5954†							3573				
		male	3524†							2735				
ScNT	Renthal	male	6328*	592	1640	599			1083	1892		1250	746	
	Wang	male	692		516	372		21		48	63	246		
SpNT	Avraham	female	6*						225					
	Renthal	male	6328*	1051	1088	1307	1111	1117	1374	621				
Exrtra control	Techameena_SS3	female	281											
		male	193											
	Parpaite	male	24											
	Sharma	female	9523											

b TG: All cells

Conditions	Source	Sex	Control	1h	1.5h	6h	1d	7d
PBS	Yang_RNA	female	10810*	6272				
		male	20190*	7638				
CFA	Liu	male	2045					1191
CSD	Yang_RNA	male	20190*		3858	1926		
IS	Yang_RNA	female	10810*	9659				
		male	20190*	6559		1943	1793	
Extra control	Jia	female	6142					
	Techameena	female	78					
		male	46					
	Nguyen	female	4386					
		male	122					
	Yang_ATAC	male	736					
		pooled	2444					

TG: Nociceptors

Conditions	Source	Sex	Control	1h	1.5h	6h	1d	7d
PBS	Yang_RNA	female	45*	317				
		male	5866*	353				
CFA	Liu	male	30					27
CSD	Yang_RNA	male	5866*		1512	1074		
IS	Yang_RNA	female	45*	184				
		male	5866*	660		718	797	
Extra control	Jia	female	136					
	Techameena	female	53					
		male	33					
	Nguyen	female	2174					
		male	78					
	Yang_ATAC	male	367					
		pooled	998					

* Redundance cells as control

† Sham at 7day as control

Supplementary Fig. 3: the atlases content.

a Tables of cell numbers from different injury models, timepoints, sexes, and data sources in iPainDRG from all cell types (left), and nociceptors (right). **b** Tables of cell numbers from different injury models, timepoints, sexes, and data sources in iPainTG from all cell types (left), and nociceptors (right).

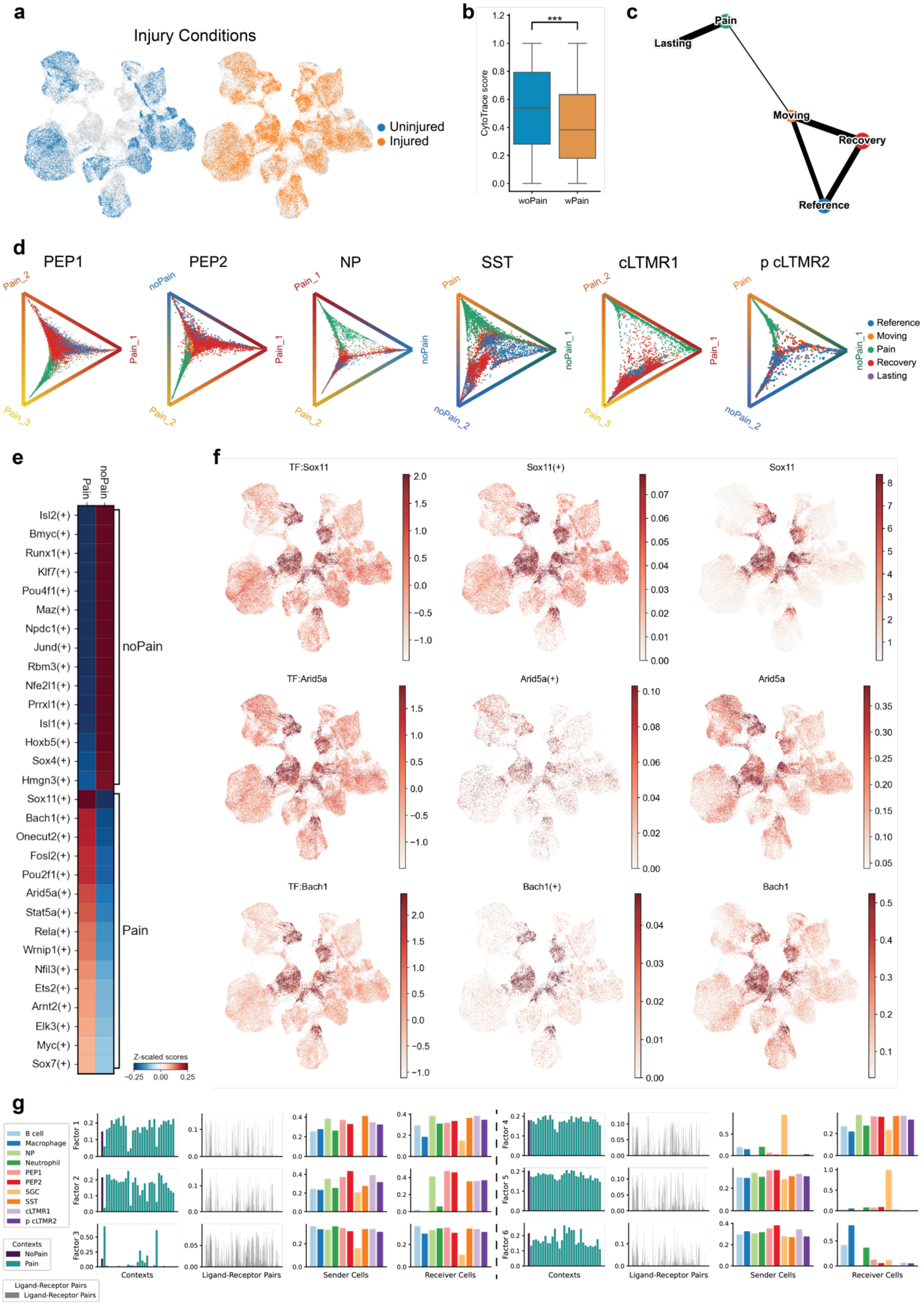
a
Injury Conditions
Uninjured
Injured
b
CytoTrace score
***
woPain
wPain
c
Pain
Lasting
Moving
Recovery
Reference
d
PEP1
PEP2
NP
SST
cLTMR1
p cLTMR2
Reference
Moving
Pain
Recovery
Lasting
e
Pain
noPain
Isl2(+)
Bmyc(+)
Runx1(+)
Klf7(+)
Pou4f1(+)
Maz(+)
Npdc1(+)
Jund(+)
Rbm3(+)
Nfe2l1(+)
Prrxl1(+)
Isl1(+)
Hoxb5(+)
Sox4(+)
Hmgn3(+)
Sox11(+)
Bach1(+)
Onecut2(+)
Fosl2(+)
Pou2f1(+)
Arid5a(+)
Stat5a(+)
Rela(+)
Wrnip1(+)
Nfil3(+)
Ets2(+)
Arnt2(+)
Elk3(+)
Myc(+)
Sox7(+)
Z-scaled scores
f
TF:Sox11
Sox11(+)
Sox11
TF:Arid5a
Arid5a(+)
Arid5a
TF:Bach1
Bach1(+)
Bach1
g
B cell
Macrophage
NP
Neutrophil
PEP1
PEP2
SGC
SST
cLTMR1
p cLTMR2
Contexts
NoPain
Pain
Ligand-Receptor Pairs
Ligand-Receptor Pairs
Factor 1
Factor 2
Factor 3
Factor 4
Factor 5
Factor 6
Contexts
Ligand-Receptor Pairs
Sender Cells
Receiver Cells

Supplementary Fig. 4: Analysis of pain sensing neurons from DRG

a UMAP plots of neurons from nociceptive lineage highlight by the respective pain conditions; left, uninjured samples; right, injured samples. **b** A boxplot comparing the CytoTrace score of neurons from the nociceptive lineage from different injury conditions with asterisk to denote the significant level (*** P=0.00013, one-sided t-test). **c** Plots of PAGA graphs with threshold equal 0.166. **d** Circular plots of cells from different subtypes within the nociceptive lineage colored by the pain dynamics. **e** A matrix plot representing the top 15 regulons from each injury condition of the neurons from the nociceptive lineage. **f** UMAP plots where each row representing the transcription activity, regulon activity, and gene expression profile (from left to right) of Sox11, Arid5a, and Bach1 (from top to bottom). **g** Plot of context loading, and degree of communication captured by different factors after tensor decomposition analysis. Plots of context score by combinatorial conditions, that includes nociceptors' subtypes, satellite glia cells and subtypes of immunes cells, the different pain models, time points. The analysis is run comparing the Reference state to non-Reference state (Moving, Pain, Recovery and Lasting). context score of all ligand receptor pairs identified. Six different factors were identified with factor 3 showing the most difference for most pain models. Box plots indicate median at center, upper and lower quartiles at the bounds of box, whiskers are at minima and maxima.

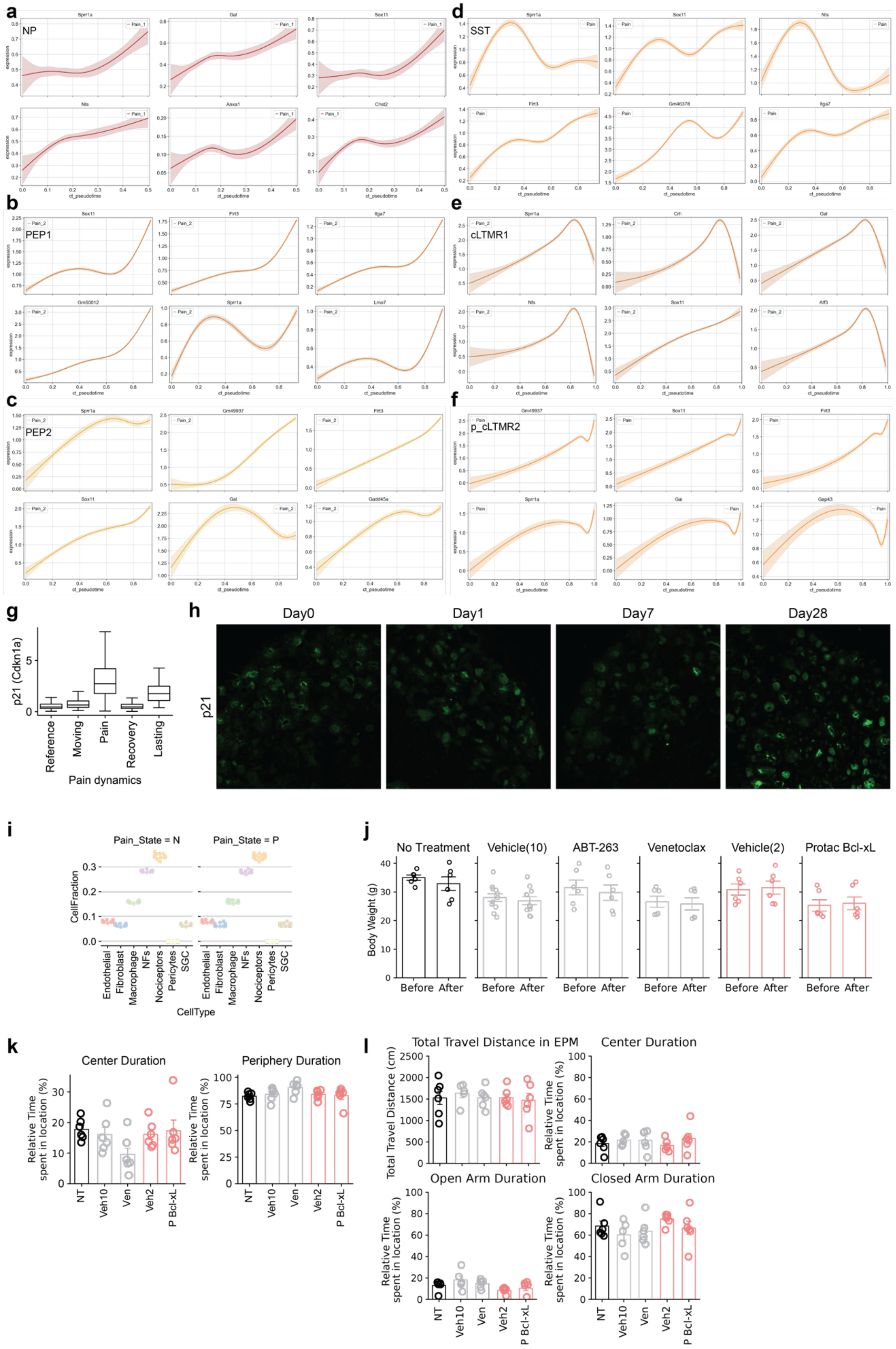
a
NP
b
PEP1
c
PEP2
d
SST
e
cLTMR1
f
p_cLTMR2
g
p21 (Cdkn1a)
Reference
Moving
Pain
Recovery
Lasting
Pain dynamics
h
Day0
Day1
Day7
Day28
p21
i
Pain_State = N
Pain_State = P
CellFraction
Endothelial
Fibroblast
Macrophage
NFs
Nociceptors
Pericytes
SGC
CellType
j
No Treatment
Vehicle(10)
ABT-263
Venetoclax
Vehicle(2)
Protac Bcl-xL
Body Weight (g)
Before After
k
Center Duration
Periphery Duration
Relative Time spent in location (%)
NT
Veh10
Ven
Veh2
P Bcl-xL
l
Total Travel Distance in EPM
Center Duration
Open Arm Duration
Closed Arm Duration
Total Travel Distance (cm)

Supplementary Fig. 5: supplementary to Fig. 2, Fig. 3, Fig. 4 and Fig. 5

a-f The gene expression trend of the top 6 driver genes from different nociceptive subtypes which are NP (**a**), PEP1 (**b**), PEP2 (**c**), SST (**d**), cLTMR1 (**e**), p_cLTMR2 (**f**). **g** Box plots of the expression of Cdkn1a (p21) by the microstates pain dynamics. **h** p21 staining images of DRG before and after injury in mice with CCI model, from 0day (control) up to 28day. **i** Swarm plots of the fraction of cell type corresponding to each bulk RNA-seq sample split by known pain conditions **j** The bar plots of mean values +/- SEM from the mice body weight before and after treatment. **k** Bar plots of mean values +/- SEM of results from open field test (ns P>0.05, two-sided t-test). **l** Bar plots of mean values +/- SEM of results from elevated plus maze test (ns P>0.05, two-sided t-test). Box plots indicate median at center, upper and lower quartiles at the bounds of box, whiskers are at minima and maxima. Note on abbreviation, NT: No Treatment; Veh10: Vehicle (10 doses of DMSO4); Veh2: Vehicle (2 doses of DMSO); Ven: Venetoclax; and P Bcl-xL: Protac Bcl-xL. Source data are provided as a Source Data file.
