# Supplementary material for: The single-cell transcriptomic atlas iPain identifies senescence of nociceptors as a therapeutical target for chronic pain treatment
Source: Nat Commun. 2024 Oct 4;15:8585. doi: 10.1038/s41467-024-52052-8 (PMC11450014; doi:10.1038/s41467-024-52052-8)
Supplement: Supplementary file 3 — Description of Additional Supplementary Files [file 41467_2024_52052_MOESM3_ESM.docx]

**Description of Additional Supplementary Data**

**Supplementary Data 1:** Sample level metadata of the single-cell/nuclei sequencing in DRG and TG

**Supplementary Data 2:** Cell type marker genes computed with differential expression method from scANVI model.

**Supplementary Data 3:** Cell type marker peaks computed with two-sided Wilcoxon rank sum test and p-values were adjusted with Benjamini-Hochberg for multiple hypothesis testing. Only genes with adjusted p-values less than 0.05 and log2 fold-change greater than zero in each group were kept.

**Supplementary Data 4:** Differentially expressed genes between “woPain” and “wPain” groups. The test was done with two-sided Wilcoxon rank sum test and p-values were adjusted with Benjamini-Hochberg for multiple hypothesis testing. Only genes with adjusted p-values less than 0.05 and log2 fold-change greater than zero in each group were kept.

**Supplementary Data 5:** List of driver genes toward Pain for each subtype of the nociceptive lineage neurons. The driver genes were calculated by correlating expression values with fate probabilities with Fisher transformation. P-values were adjusted with Benjamini-Hochberg for multiple hypothesis testing.

**Supplementary Data 6:** List of ligand-receptor pairs involved in the cell-to-cell communication process between different subtypes of nociceptive lineage neurons, immune cells, and satellite glial cells. The list was computed by multiple methods provided by LIANA package.

**Supplementary Data 7:** Table containing genes within the SenMayo geneset for mouse and human.

**Supplementary Data 8:** Table containing SenMyo, GenAge, and CellAge genesets.
