# Supplementary material for: The single-cell transcriptomic atlas iPain identifies senescence of nociceptors as a therapeutical target for chronic pain treatment
Source: Nat Commun. 2024 Oct 4;15:8585. doi: 10.1038/s41467-024-52052-8 (PMC11450014; doi:10.1038/s41467-024-52052-8)
Supplement: Supplementary file 12 — Reporting Summary [file 41467_2024_52052_MOESM12_ESM.pdf]

nature portfolio

Corresponding author(s): Saida Hadjab

Last updated by author(s): Jul 19, 2024

Reporting Summary

Nature Portfolio wishes to improve the reproducibility of the work that we publish. This form provides structure for consistency and transparency in reporting. For further information on Nature Portfolio policies, see our Editorial Policies and the Editorial Policy Checklist.

Statistics

For all statistical analyses, confirm that the following items are present in the figure legend, table legend, main text, or Methods section.

n/a	Confirmed	
☐	☒	The exact sample size (*n*) for each experimental group/condition, given as a discrete number and unit of measurement
☒	☐	A statement on whether measurements were taken from distinct samples or whether the same sample was measured repeatedly
☐	☒	The statistical test(s) used AND whether they are one- or two-sided<br>*Only common tests should be described solely by name; describe more complex techniques in the Methods section.*
☐	☒	A description of all covariates tested
☒	☐	A description of any assumptions or corrections, such as tests of normality and adjustment for multiple comparisons
☐	☒	A full description of the statistical parameters including central tendency (e.g. means) or other basic estimates (e.g. regression coefficient) AND variation (e.g. standard deviation) or associated estimates of uncertainty (e.g. confidence intervals)
☐	☒	For null hypothesis testing, the test statistic (e.g. *F*, *t*, *r*) with confidence intervals, effect sizes, degrees of freedom and *P* value noted<br>*Give P values as exact values whenever suitable.*
☒	☐	For Bayesian analysis, information on the choice of priors and Markov chain Monte Carlo settings
☒	☐	For hierarchical and complex designs, identification of the appropriate level for tests and full reporting of outcomes
☒	☐	Estimates of effect sizes (e.g. Cohen's *d*, Pearson's *r*), indicating how they were calculated

Our web collection on statistics for biologists contains articles on many of the points above.

Software and code

Policy information about availability of computer code

Data collection	10x snMultiomics: The samples were sequenced on NovaSeq6000 (NovaSeq Control Software 1.7.5/RTA v3.4.4) with a 50nt(Read1)-8nt (Index1)-24nt(Index2)-49nt(Read2) (ATAC part) and 28nt(Read1)-10nt(Index1)-10nt(Index2)-90nt(Read2) (GEX part) setup using 'NovaSeqStandard' workflow in 'S2' mode flowcell. The Bcl to FastQ conversion was performed using bcl2fastq_v2.20.0.422 from the CASAVA software suite. The quality scale used is Sanger / phred33 / Illumina 1.8+. Cell Ranger Arc v2.0.2 software was used to process the raw sequencing files to generate the count matrices.<br>Smart-seq3 express: zUMIs v2.9.7 software was used to process the raw sequencing files to generate the count matrices.<br>Brightfield images were acquired using the Zeiss Axio Imager.Z2.

Data analysis	Single-cell/nucleus omics analysis: The code for data analysis is available on https://github.com/PrachTecha/Hadjab_iPain and based on open-source python packages.<br>ImageJ version 1.52h software (NIH) was used for the quantification of senescence-associated beta-galactosidase staining with help of the Colour Deconvolution plugin.<br>Single-cell/nuclei analysis was done with Python3 (v3.10.10) using packages and versions listed below :<br>Scanpy(v1.9.3)<br>scvi-tools(v0.20.3)<br>LIANA(v0.1.8)<br>CellRank(v2.0.0)<br>scGLUE (v0.3.2)<br>Statistical analysis was done with SciPy version 1.10.1 Python package.

For manuscripts utilizing custom algorithms or software that are central to the research but not yet described in published literature, software must be made available to editors and reviewers. We strongly encourage code deposition in a community repository (e.g. GitHub). See the Nature Portfolio guidelines for submitting code & software for further information.

Data

Policy information about availability of data

All manuscripts must include a data availability statement. This statement should provide the following information, where applicable:

- Accession codes, unique identifiers, or web links for publicly available datasets
- A description of any restrictions on data availability
- For clinical datasets or third party data, please ensure that the statement adheres to our policy

The sequencing data and count matrices generated from this study have been deposited in the Gene Expression Omnibus (GEO) database under accession code GSE253345. The interactive visualization of the atlases can be accessed through CELLxGENE platform from Chan Zuckerberg Initiative (iPain :https://cellxgene.cziscience.com/collections/03608e22-227a-4492-910b-3cb3f16f952e).

Research involving human participants, their data, or biological material

Policy information about studies with human participants or human data. See also policy information about sex, gender (identity/presentation), and sexual orientation and race, ethnicity and racism.

Reporting on sex and gender	*Use the terms sex (biological attribute) and gender (shaped by social and cultural circumstances) carefully in order to avoid confusing both terms. Indicate if findings apply to only one sex or gender; describe whether sex and gender were considered in study design; whether sex and/or gender was determined based on self-reporting or assigned and methods used.*<br>*Provide in the source data disaggregated sex and gender data, where this information has been collected, and if consent has been obtained for sharing of individual-level data; provide overall numbers in this Reporting Summary. Please state if this information has not been collected.*<br>*Report sex- and gender-based analyses where performed, justify reasons for lack of sex- and gender-based analysis.*
Reporting on race, ethnicity, or other socially relevant groupings	*Please specify the socially constructed or socially relevant categorization variable(s) used in your manuscript and explain why they were used. Please note that such variables should not be used as proxies for other socially constructed/relevant variables (for example, race or ethnicity should not be used as a proxy for socioeconomic status).*<br>*Provide clear definitions of the relevant terms used, how they were provided (by the participants/respondents, the researchers, or third parties), and the method(s) used to classify people into the different categories (e.g. self-report, census or administrative data, social media data, etc.)*<br>*Please provide details about how you controlled for confounding variables in your analyses.*
Population characteristics	*Describe the covariate-relevant population characteristics of the human research participants (e.g. age, genotypic information, past and current diagnosis and treatment categories). If you filled out the behavioural & social sciences study design questions and have nothing to add here, write "See above."*
Recruitment	*Describe how participants were recruited. Outline any potential self-selection bias or other biases that may be present and how these are likely to impact results.*
Ethics oversight	*Identify the organization(s) that approved the study protocol.*

Note that full information on the approval of the study protocol must also be provided in the manuscript.

Field-specific reporting

Please select the one below that is the best fit for your research. If you are not sure, read the appropriate sections before making your selection.

[x] Life sciences [ ] Behavioural & social sciences [ ] Ecological, evolutionary & environmental sciences

For a reference copy of the document with all sections, see nature.com/documents/nr-reporting-summary-flat.pdf

Life sciences study design

All studies must disclose on these points even when the disclosure is negative.

Sample size	Based on our own experience and similar studies, 5-6 animals are sufficient to observe significant differences among different groups for behaviour tests. For sequencing, a minimum of 3 mice per group was chosen based on similar studies. For senescence-associated beta-galactosidase staining, a total of 6 DRGs corresponding to the injury level (3 per side) were taken for analysis per mouse. No statistical methods were used to predetermine sample sizes.<br>10x single-nucleus Multiomics: 3 mice were pooled together to generate the sequencing data per group for a total of 12 mice in total.<br>Smart-seq3xpress single-nucleus RNA-seq: 4 mice were included to generate data.<br>Senescence-associated beta-galactosidase staining: 2 mice were included for each time point for a total of 10 mice.<br>Drug treatment and behavior tests: 11 mice were included where 5 mice were in control group and 6 mice were in senolytic treatment group for behavior test.
Data exclusions	For single-cell multiomics, cells with less than log-base e of 4, and more than log-base e of 9 were removed. Cells with more than 1.5% mitochondrial gene count were also removed. For smart-seq3 express, cells with less than 1000 unique genes were excluded.
Replication	10x single-nucleus Multiomics: The nuclei were pooled from 3 mice for each group to generate the data with 4 groups in total.<br>Smart-seq3xpress single-nucleus RNA-seq: The nuclei were pooled from 4 mice to generate 2 plates of smart seq3 express data.<br>Senescence-associated beta-galactosidase staining: There were two replicates for each time point with 5 time points in total.<br>The chronic pain model and drug characteristics have been confirmed by numerous different investigators with different paradigms. The pain model have been successfully used multiple times within our lab.
Randomization	Mice were allocated to different experimental groups based on their randomly assigned ID by the experimenter with considerations for matching sex and age.
Blinding	No blinding was done for single-nucleus data. Blinding experiment was done for behavior test where the experimenter was not fully aware of the treatment groups.

Reporting for specific materials, systems and methods

We require information from authors about some types of materials, experimental systems and methods used in many studies. Here, indicate whether each material, system or method listed is relevant to your study. If you are not sure if a list item applies to your research, read the appropriate section before selecting a response.

Materials & experimental systems

n/a	Involved in the study	
☐	☒	Antibodies
☒	☐	Eukaryotic cell lines
☒	☐	Palaeontology and archaeology
☐	☒	Animals and other organisms
☒	☐	Clinical data
☒	☐	Dual use research of concern
☒	☐	Plants

Methods

n/a	Involved in the study	
☒	☐	ChIP-seq
☐	☒	Flow cytometry
☒	☐	MRI-based neuroimaging

Antibodies

Antibodies used	For FACS: anti-NeuN PE conjugated antibody (1:500 FCMAB317PE, Merck)
Validation	The antibody was validated by the manufacturer.

Animals and other research organisms

Policy information about studies involving animals; ARRIVE guidelines recommended for reporting animal research, and Sex and Gender in Research

Laboratory animals	All animals included in this study were wildtype C57BL/6 mice, purchased originally from the Jackson Laboratory and maintained in house. Groups of Young (8 weeks) and Old (34–62 weeks) mice of both sexes were included within the atlas while 17-27 weeks old mice of both sexes were used in drug administration followed by behavioral test processes and SA-β-Galactosidase staining. Animals were group-housed, with food and water ad libitum, under 12 h light–dark cycle conditions, under standardized condition in the animal facility of Karolinska Institutet.
Wild animals	No wild animals were used in this study.

Reporting on sex	Animals of either sex were used in this study.
Field-collected samples	No field-collected animals were used in this study.
Ethics oversight	All animal work was performed in accordance with the national guidelines and approved by the local ethics committee of Stockholm, Stockholms Norra djurförsöksetiska nämnd.

Note that full information on the approval of the study protocol must also be provided in the manuscript.

Plants

Seed stocks	*Report on the source of all seed stocks or other plant material used. If applicable, state the seed stock centre and catalogue number. If plant specimens were collected from the field, describe the collection location, date and sampling procedures.*
Novel plant genotypes	*Describe the methods by which all novel plant genotypes were produced. This includes those generated by transgenic approaches, gene editing, chemical/radiation-based mutagenesis and hybridization. For transgenic lines, describe the transformation method, the number of independent lines analyzed and the generation upon which experiments were performed. For gene-edited lines, describe the editor used, the endogenous sequence targeted for editing, the targeting guide RNA sequence (if applicable) and how the editor was applied.*
Authentication	*Describe any authentication procedures for each seed stock used or novel genotype generated. Describe any experiments used to assess the effect of a mutation and, where applicable, how potential secondary effects (e.g. second site T-DNA insertions, mosiacism, off-target gene editing) were examined.*

Flow Cytometry

Plots

Confirm that:

- [x] The axis labels state the marker and fluorochrome used (e.g. CD4-FITC).
- [x] The axis scales are clearly visible. Include numbers along axes only for bottom left plot of group (a 'group' is an analysis of identical markers).
- [x] All plots are contour plots with outliers or pseudocolor plots.
- [x] A numerical value for number of cells or percentage (with statistics) is provided.

Methodology

Sample preparation	DRG nuclei were dissociated as mentioned in Methods section in Fluorescent-activated nuclei sorting paragraph.
Instrument	BD instruments. BD FACSDiva 8.0.2 and BD Influx were used.
Software	FlowJo v10 (BD) and BD FACS Sortware 1.2.0/142/Utopex 1.2.0.108
Cell population abundance	The cell population abundance of the DAPI+ singlet cells were 52.8% and 51% from the total population
Gating strategy	DAPI-positive single nuclei were selected based on observed intensity and ratio of SSC-H/SSC-A. NeuN-positive nuclei were further selected to gate neuronal cells.

- [x] Tick this box to confirm that a figure exemplifying the gating strategy is provided in the Supplementary Information.
